# Evidence for Antisense Transcription Associated with MicroRNA Target mRNAs in Arabidopsis

**DOI:** 10.1371/journal.pgen.1000457

**Published:** 2009-04-17

**Authors:** Qing-Jun Luo, Manoj P. Samanta, Fatih Köksal, Jaroslav Janda, David W. Galbraith, Casey R. Richardson, Fangqian Ou-Yang, Christopher D. Rock

**Affiliations:** 1 Department of Biological Sciences, Texas Tech University, Lubbock, Texas, United States of America; 2 Systemix Institute, Los Altos, California, United States of America; 3 Department of Mathematics and Statistics, Texas Tech University, Lubbock, Texas, United States of America; 4 BIO5 Institute and Department of Plant Science, University of Arizona, Tucson, Arizona, United States of America; 5 Department of Computer Science, Texas Tech University, Lubbock, Texas, United States of America; The Salk Institute for Biological Studies, United States of America

## Abstract

Antisense transcription is a pervasive phenomenon, but its source and functional significance is largely unknown. We took an expression-based approach to explore microRNA (miRNA)-related antisense transcription by computational analyses of published whole-genome tiling microarray transcriptome and deep sequencing small RNA (smRNA) data. Statistical support for greater abundance of antisense transcription signatures and smRNAs was observed for miRNA targets than for paralogous genes with no miRNA cleavage site. Antisense smRNAs were also found associated with *MIRNA* genes. This suggests that miRNA-associated “transitivity” (production of small interfering RNAs through antisense transcription) is more common than previously reported. High-resolution (3 nt) custom tiling microarray transcriptome analysis was performed with probes 400 bp 5′ upstream and 3′ downstream of the miRNA cleavage sites (direction relative to the mRNA) for 22 select miRNA target genes. We hybridized RNAs labeled from the smRNA pathway mutants, including *hen1-1*, *dcl1-7*, *hyl1-2*, *rdr6-15*, and *sgs3-14*. Results showed that antisense transcripts associated with miRNA targets were mainly elevated in *hen1-1* and *sgs3-14* to a lesser extent, and somewhat reduced in *dcl11-7*, *hyl11-2*, or *rdr6-15* mutants. This was corroborated by semi-quantitative reverse transcription PCR; however, a direct correlation of antisense transcript abundance in *MIR164* gene knockouts was not observed. Our overall analysis reveals a more widespread role for miRNA-associated transitivity with implications for functions of antisense transcription in gene regulation. HEN1 and SGS3 may be links for miRNA target entry into different RNA processing pathways.

## Author Summary

Antisense transcription is a pervasive but poorly understood phenomenon in a wide variety of organisms. We have found evidence for a novel source of antisense transcription in *Arabidopsis thaliana* associated with miRNA targets via computational analyses of published whole-genome tiling microarray data, deep sequencing smRNA datasets, and from custom high-resolution (3 nt) tiling microarray analysis. Our data show increased antisense transcription for select miRNA targets in the *hua enhancer1-1* (*hen1-1*), a smRNA methyltransferase mutant, and the *suppressor of gene silencing3-14* (*sgs3-14*) mutant that affects post-transcriptional gene silencing and leaf development. Additional results suggest that miRNA targets and *MIRNA* genes are subject to the activities of both the miRNA and RNA silencing pathways in which HEN1 and SGS3 may represent associated nodes. The analysis of sense–antisense transcripts using high-resolution tiling microarrays and genetic mutants provides a precise and sensitive means to study epigenetic activities. Our method of mining expression data of plant miRNAs targets and smRNAs is potentially applicable to the identification of epigenetic targets in metazoans, where computational methods for prediction of miRNAs and their targets lack power because of sequence degeneracy, and to identify loci producing antisense transcripts by triggers other than miRNA-directed cleavage.

## Introduction

Non-coding genes, such as those producing miRNAs and small interfering RNAs (siRNAs), are key components of gene expression in eukaryotes, forming a regulatory network superimposed on the central dogma of molecular biology [1],[2],[3]. miRNAs are expressed through nucleolytic maturation of hairpin precursors transcribed by RNA Polymerase II or III [4],[5]. siRNAs are derived either from endogenous transcripts that form perfect double-stranded RNA (dsRNA) structures, or from transcripts of transgenes, viral genomes and protein-coding genes including miRNA targets that act as substrates for the RNA-induced silencing complex (RISC). Both classes of smRNAs are involved in post-transcriptional gene regulation in plants, fungi and animals [1],[3]. miRNAs bind to target RNA transcripts and guide their cleavage (mostly for plants) or act to prevent translation [6],[7],[8]. siRNAs act via a similar mechanism of cleavage of their target genes, but they also can direct genomic DNA methylation and chromatin remodeling [9]. It is estimated that at least 20–30% of all human genes may be post-transcriptionally regulated by miRNAs [10].

Transcriptome profiling experiments have demonstrated the extensive presence of endogenous antisense transcripts both in plants and animals [11],[12],[13], but the mechanisms and significance of such transcriptional activities are still not clear. One hypothesis is that miRNAs trigger the production of the antisense transcripts from their cognate sense transcripts, which in turn generate smRNAs for gene silencing, in a phenomenon known as transitivity [14],[15],[16]. This hypothesis is derived from several indirect and direct lines of evidence. Parizotto *et al.* [17] observed that stringent mutations within miRNA target sequences can prevent cleavage, but may not entirely prevent transitivity through siRNAs. This suggests that miRNAs may have additional activities or determinants in post-transcriptional regulation that are independent of cleavage. Furthermore, miRNAs are known to generate *trans*-acting siRNAs (ta-siRNAs), a subclass of smRNAs, through antisense transcription associated with RNA DEPENDENT RNA POLYMERASE 6 (RDR6) [14],[15],[18],[19]. ta-siRNAs differ from classical siRNAs by silencing mRNAs unrelated to their primary transcript. For example, ta-siRNAs target pentatricopeptide repeat-containing genes (*PPR*) of unknown function and transcription factors involved in vegetative development and organ polarity [18],[19].

A more direct line of evidence for miRNA target-associated transitivity comes from several studies that characterized antisense transcripts or smRNAs for miRNA targets, including *SPL3*, *SPL10*, *TIR1*, *HAP2C* and a clade of *PPR* genes [15],[16],[20],[21]. Those antisense transcripts appear to function in transitive silencing involving RDRs and miRNA/siRNA processing [16],[21]. Axtell *et al.* [15] described a mechanism for transitivity of some miRNA target genes, including *PPR* and *TAS3*. These transcripts have a second, cryptic miRNA binding site that can trigger siRNA production without cleavage. It has also been speculated that methylation of miRNAs at the 3′-terminal hydroxyl group by HEN1 may serve to counteract the antisense transcription activity primed possibly by unmethylated miRNAs [22]. However, the known cases of transitivity associated with miRNA target genes to date are few and limited to RDR6-dependent production of siRNAs downstream (direction relative to the coding strand) of the miRNA binding site in the plant *Arabidopsis thaliana* [15],[16],[23].

In work presented here, we show that antisense transcription of miRNA targets and *MIRNA* genes in the model plant Arabidopsis is more prevalent than previously observed. Our findings were guided by statistical analyses of extant whole-genome and smRNA transcriptome databases. Antisense transcripts were characterized by RNA transcript profiling of smRNA pathway-defective mutants with a custom high-resolution (3 n.t.) microarray, and their existence was corroborated by semi-quantitative reverse transcription PCR (qRT-PCR). Most antisense transcripts near the miRNA target sites were elevated in *hen1-1* and a few were also upregulated in the *sgs3-14* mutant, which affects post-transcriptional gene silencing and leaf development [14],[24]. Our findings suggest that HEN1 and SGS3 may work in the same process/step to suppress synthesis or stability of miRNA target-associated antisense transcripts, which might serve as a link between miRNA and RNA silencing pathways.

## Results

### MPSS Signatures of Antisense Transcripts Are Associated with MiRNA Targets

The digital and normalized nature of Massively Parallel Signature Sequencing (MPSS) data enables one to mathematically analyze the expression relationship of all transcriptional signatures (e.g. sense and antisense) both within and between samples. We analyzed the abundances of sense and antisense signatures for miRNA targets from the MPSS Plus Database (http://mpss.udel.edu/at) [25],[26]. A scalar value was calculated representing the abundance of antisense signatures divided by that of total signatures for a given gene. Thirteen out of the total seventeen MPSS libraries showed a higher percentage of normalized antisense signatures associated with the experimentally validated miRNA targets (n = 94, Tables 1, S1 and S2) than for paralogous non-targets (n = 140). The paralog genes included fourteen experimentally verified non-miRNA-targets [19],[27] and were chosen as biological controls based on the presence of a remnant pseudo-miRNA binding site that presumably does not associate with a miRNA because of sequence divergence (see Materials and Methods). For the six inflorescence libraries (the *INF*, *INS*, *AP1*, *SAP*, *AP3* and *AGM* samples in Table 1), five had a greater abundance of normalized antisense signatures for validated targets than did paralogs, and the higher expression in the *INS* library was significant (*P*<0.05, one-sided Student's t-test, equal variance model). Other tissues, including callus, leaf, root, silique and seedling (the *CAS*, *LES*, *ROS*, *SIS*, *GSE* libraries in Table 1) showed the correlation of higher antisense expression for validated targets as well, arguing against a tissue-specific bias for these antisense transcripts despite high levels of miRNAs in flowers [20]. It is noteworthy that all twelve “signature method” MPSS libraries (labeled by † in Table 1) gave higher normalized antisense signatures for validated miRNA targets, whereas four out of five of the “classic method” libraries did not (labeled by * in Table 1), raising questions about possible technical bias in the classic MPSS datasets as noted (http://mpss.udel.edu/at/). Discounting the “classic method” signature data, a combined statistical analysis of the “signature method” libraries showed that validated miRNA targets have significantly higher normalized antisense transcript expression than their paralog genes (*P*<0.05, one-sided Student's t-test, equal variance model, Tables 1 and S2). The *TAS1–TAS4* genes are targets of miR173, miR390 or miR828 and they require antisense transcription to generate ta-siRNAs [19],[28]. When these target genes were removed from the analysis, the average normalized antisense signature abundance for the validated miRNA targets in all 17 libraries increased (data not shown), demonstrating that antisense transcription of non-*TAS* miRNA target genes is substantial. Our observations suggest that mechanisms similar to those operating in the production of ta-siRNAs may also act on many bona fide miRNA targets previously concluded to be intransitive [20].

**Table 1:** Normalized fraction of antisense transcript signature abundance of validated miRNA targets, predicted targets and paralogous non-targets from the Massively Parallel Signature Sequencing (MPSS) Plus Database^a^.

	CAF*	CAS†	INF*	INS†	AP1†	SAP†	AP3†	AGM†	LEF*	LES†	S04†	S52†	ROF*	ROS†	SIF*	SIS†	GSE†	Combined^g^
Validated Targets^b^	0.021	0.016	0.021	0.065	0.075	0.066	0.071	0.054	0.047	0.070	0.034	0.036	0.041	0.060	0.025	0.021	0.148	0.060
Predicted Targets^c^	0.023	0.018	0.008	0.030	0.034	0.034	0.021	0.022	0.021	0.025	0.021	0.019	0.019	0.025	0.014	0.015	0.059	0.027
Paralogs^d^	0.027	0.010	0.045	0.029	0.057	0.049	0.059	0.033	0.025	0.04	0.026	0.034	0.050	0.052	0.027	0.008	0.124	0.044
Differential^e^	low	high	low	high	high	high	high	high	high	high	high	high	low	high	low	high	high	high
*P*-value^f^	0.346	0.244	0.135	0.026	0.214	0.232	0.308	0.166	0.107	0.100	0.332	0.468	0.352	0.370	0.449	0.166	0.269	0.007

a Sum of abundance for mRNA antisense signatures (classes 3 and 6, transcript per million) was collected from the MPSS Plus Database (http://mpss.udel.edu/at/, Table S2). Normalized data for all 17 libraries in MPSS database was obtained by dividing the abundance of mRNA antisense signatures for the total signatures associated with each gene, and the average for total genes in each set is presented here (percentage of mRNA antisense signatures in total signatures/locus). The abbreviation for all 17 libraries is as follows: CAF, CAS: actively growing callus; INF, INS: inflorescence; AP1: *apetela1-10* mutant inflorescence; SAP: *superman/apetela1* mutant inflorescence; AP3: *apetela3-6* mutant inflorescence; AGM: *agamous* mutant inflorescence (all inflorescence samples were collected from immature buds of mixed stages); LEF, LES: untreated leaves of 21 days; S04: leaves of 4 hr after salicylic acid treatment; S52: leaves of 52 hr after salicylic acid treatment; ROF, ROS: untreated root of 21 days; SIF, SIS: silique of 24 to 48 hr post-fertilization; GSE: germinating seedlings. *: Data from the classic MPSS method; ^†^: data from the signature MPSS method.

b n = 94.

c n = 283.

d n = 140.

e The difference between validated miRNA targets and paralogous non-targets for the percentage of mRNA antisense signatures in total signatures.

f Individual *P*-value of the Student's t-test analysis (one-sided, equal variance model) for the percentages of mRNA antisense signatures in total signatures between validated miRNA targets and paralogous non-targets in each library.

g Combined *P*-value for the percentages of mRNA antisense signatures between validated miRNA target versus paralogous non-targets in all signature libraries (labeled by †).

### Whole Genome Tiling Microarray Transcriptome Data Reveal a Correlation between Antisense Transcription and MiRNA Target Sites

**Figure 1:**
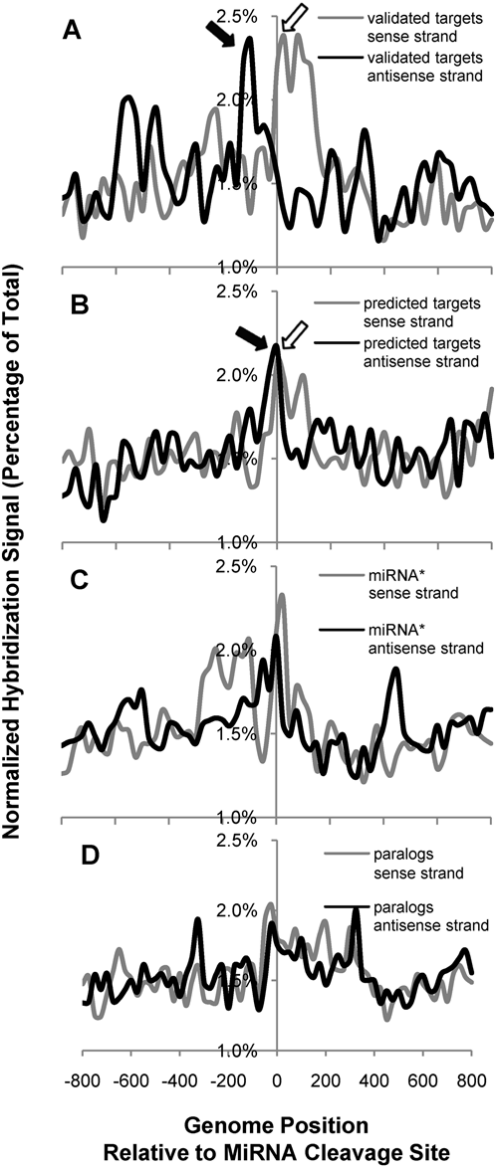
Average topology of sense and antisense transcript signals spanning miRNA target sites. (A) Validated miRNA targets (n = 78); (B) Predicted miRNA targets (n = 188); (C) miRNA genes (n = 159); (D) paralogous non-targets (n = 120). Data was collected from two published whole genome tiling microarray experiments with five samples from Arabidopsis flowers, leaves, roots, and two suspension cultures [11],[13]. For validated and predicted targets, each data point on the plot is the average of the normalized total signal from five tissue samples spanning 800 n.t. upstream and downstream of the validated or predicted miRNA cleavage sites. For *MIRNA* genes or paralogous non-targets, data for the same length of region spanning miRNA* sites or pseudo-binding sites was plotted. Signals on the sense strand are indicated by gray line and open arrow, while antisense signals are displayed by black line and black arrow. In panel A, antisense signals within the 200 n.t. range upstream (black arrow) and sense signals within the 200 n.t. range downstream (open arrow) of the miRNA cleavage site (coordinate 0 on x-axis) for validated targets have significantly higher signal intensity than elsewhere on the plot and than those in the same region of paralogs (95% confidence interval, see Tables S3 and S4). In panel B, antisense signals within the 200 n.t. range upstream of the predicted miRNA cleavage site (black arrow) is also statistically higher than those in the same region of paralogs.

The high percentage of MPSS normalized antisense signatures for the validated miRNA targets prompted us to perform a systematic survey of antisense transcription for miRNA targets and *MIRNA* genes. We collectively plotted the sense and antisense transcript abundance as a function of miRNA cleavage sites for validated targets (n = 78), predicted targets (n = 188), non-target paralogs (n = 120), and the miRNA* sites of *MIRNA* genes (as potential cleavage sites by miRNAs [29], n = 159) (See Text S1 and Table S3). This analysis excluded *PPR* genes, *ARGONAUTE1 (AGO1)*, *DICER-LIKE1 (DCL1)* (which harbors *MIR838* within intron 14), and the *ARF2/3/4* targets of ta-siRNAs derived from miR390 cleavage of *TAS3* (AT3G17185), because these are reported evidence for miRNA target-associated transitivity [16],[20],[23],[28]. Figure 1 presents the sense and antisense strand expression as a function of the miRNA target sites. We identified a pair of expression peaks associated with validated miRNA targets flanking the miRNA cleavage site on the sense and antisense strands, which was not seen in paralogs relative to their cryptic pseudo miRNA-binding sites (Figure 1A and D). For the validated targets, an expression peak was observed immediately downstream of the miRNA cleavage site on the sense strand (Figure 1A open arrow, referred to as “downstream sense signal” hereafter). This could be a manifestation of higher stability of the 3′ RISC cleavage fragment for miRNA target mRNAs. This interpretation is consistent with previous reports describing the accumulation of 3′ endonucleolytic cleavage products of miRNA targets by Northern blot [6], reverse genetic analysis [30], and deep sequencing of non-capped polyA^+^ “degradome” libraries [31],[32],[33]. Associated with this downstream sense signal was an additional peak of transcription signal located in a 200 n.t. region upstream of the miRNA target sites on the antisense strand (Figure 1A black arrow, referred to as “upstream antisense signal” hereafter). Figure S1 provides additional examples of this phenomenon for high downstream-sense coupled to corresponding upstream-antisense transcript signals around the miRNA binding site for twelve different miRNAs, in which target genes also produce smRNAs. For the predicted miRNA targets, an expression pattern similar to that of validated targets was observed spanning the predicted cleavage sites (Figure 1B, open arrow for downstream sense signal and black arrow for upstream antisense signal). Statistical analysis indicated that the downstream sense and upstream antisense signals were significantly higher than the average signal elsewhere on either sense or antisense strand for validated miRNA targets and predicted targets (*P*<0.01, one-sided Student's t-test, equal variance model; Table S3). The pairs of downstream sense and upstream antisense signals for the validated targets were significantly higher compared to the same region for paralogs (Table S4, 95% confidence interval calculated). In line with the recent report of miR172-mediated cleavage of the pri-miR172b transcripts [29], we observed some sense expression signals immediately downstream of the miRNA* sites of *MIRNA* genes along with some antisense expression signals immediately upstream of the miRNA* sites (Figure 1C). This implies that *MIRNA* genes may share the same process of antisense transcription with the validated miRNA targets, possibly by miRNA interaction with miRNA primary transcripts. These observations suggested a causal relationship between miRNA target site regulation and antisense transcripts of miRNA targets and *MIRNA* genes that warranted further study.

### MiRNA Target-Associated Antisense Transcripts Are Affected in SmRNA Pathway Mutants

With the confirmation by two pilot custom tiling microarray experiments that the upstream antisense expression for the validated miRNA targets was technically and biologically reproducible (see Text S1), we designed two custom 3 n.t. high resolution tiling microarrays (25mer and 36mer probe lengths; Agilent Technologies, Santa Clara, CA) to test the role of HEN1, DCL1, HYPONASTIC LEAVES1 (HYL1), RDR6 and SGS3 in production of antisense transcripts associated with validated miRNA targets. The 22 target genes on the arrays were chosen based on the presence or absence of associated smRNAs that mapped to the loci, on various amplitudes of the antisense transcription signals in published whole genome tiling microarray experiments [11],[13] (Table S3), and in order to provide a representative cross section of miRNA families. The sensitivity and precision of the custom high resolution tiling microarray to detect bona fide transcripts was evidenced by three sense strand analyses: (1) by excellent concordance of the sense strand signals of Col-0 inflorescence samples relative to the two independent whole genome tiling array transcriptome datasets (Figure S2), (2) by an absence of signals from probes corresponding to annotated introns (see Figure S2A, E, F), and (3) by the observation of reasonably good concordance for the changes in miRNA target gene sense strand expression in *hen1-1* mutant versus Ler-0 wild type between the custom tiling microarray and published data [19] using ATH1 microarrays (Figure S3).

**Figure 2:**
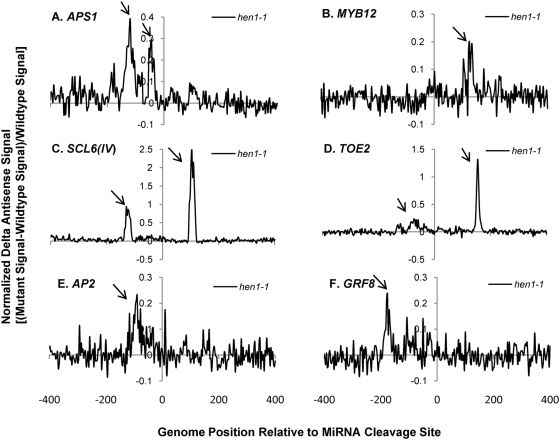
Normalized delta signals for antisense transcripts of selected validated miRNA targets showing differences between *hen1-1* versus wild type Ler-0. (A) *APS1*/AT3G22890; (B) *MYB12*/AT2G47460; (C) *SCR6(IV)*/AT4G00150; (D) *TOE2*/AT5G60120; (E) *AP2*/AT4G36920; (F) *GRF8*/AT4G24150. Each data point is the average signal of at least 3 technical samples and is represented by the difference between the signals from *hen1-1* versus Ler-0 divided by that from Ler-0 [normalized “delta” Δ signal, (mutant signal-wild type signal)/wild type signal]. The normalized delta signal is plotted as a function of probe position relative to the miRNA cleavage site (coordinate 0 on x-axis). Black arrow pinpoints the signals in the plot which were identified by probe sets containing at least 3 contiguous probes showing at least 20% differences (up or down, not both) for the normalized delta signals. The precise same region with changed signals, if any, is indicated by black arrows for other smRNA mutants in Figures 3–6.

**Figure 3 pgen-1000457-g003:**
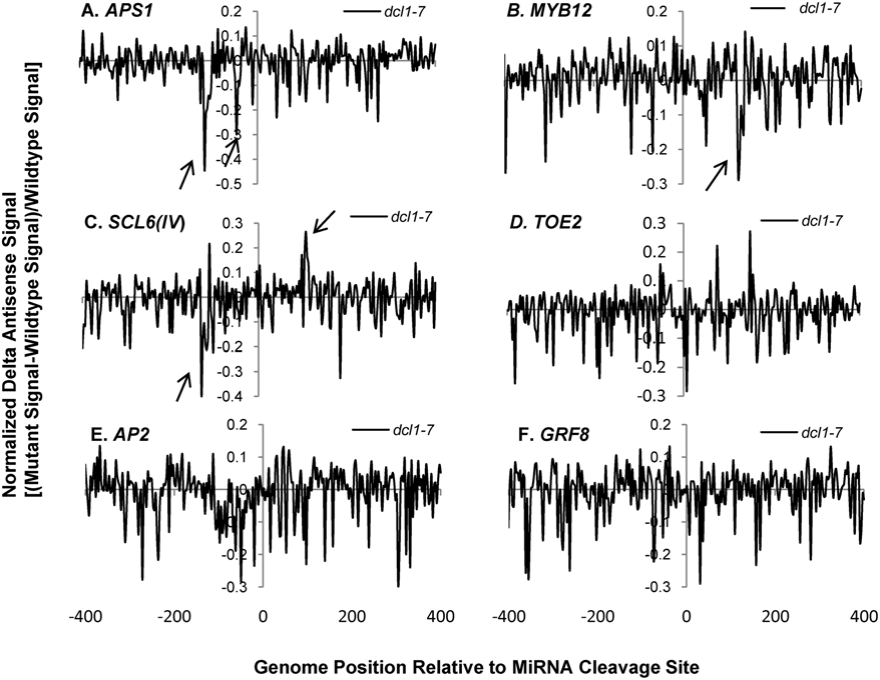
Normalized delta signals for antisense transcripts of selected validated miRNA targets showing differences between *dcl1-7* versus wild type Col-0. Refer to Figure 2 for details of legend.

**Figure 4 pgen-1000457-g004:**
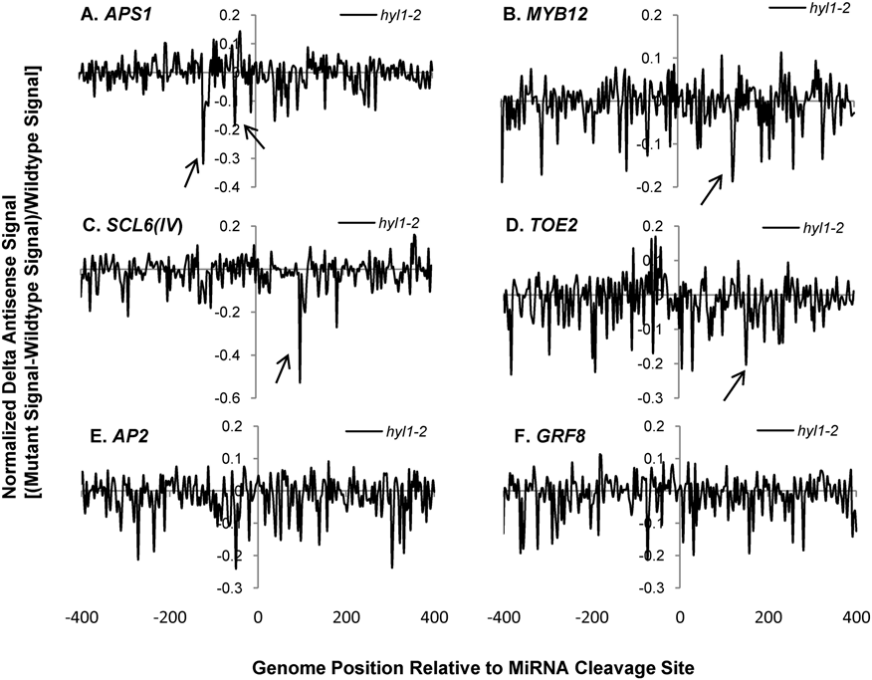
Normalized delta signals for antisense transcripts of selected validated miRNA targets showing differences between *hyl1-2* versus wild type Col-0. Refer to Figure 2 for details of legend.

Having validated the custom tiling microarray sense strand signals, the antisense signals for the miRNA targets were characterized for smRNA pathway mutants. Sixteen out of 22 genes on the microarray showed clear antisense transcription signals usually falling within 200 n.t. range upstream and/or downstream of the miRNA cleavage sites (Table S6 and Figure S4). We employed “normalized delta plots” for antisense transcripts (to facilitate gene-by-gene analyses) representing the differences between the means of signal intensities for biological and technical replicates of smRNA pathway mutants versus corresponding wild-type controls divided by the signals from wild-type. Fourteen of these sixteen genes displayed different amplitude antisense signals in at least one of the five smRNA pathway mutants *hen1-1*, *dcl1-7*, *hyl1-2*, *rdr6-15*, and *sgs3-14*. Most strikingly, the antisense signals of thirteen genes were increased in *hen1-1* mutants (Table S7). Figure 2 shows normalized delta plots for *APS1/*AT3G22890, *MYB12/*AT2G47460, *AP2/*AT4G36920, and *GRF8/*AT4G24150 antisense transcript signals which demonstrate 20–40% increases in *hen1-1* relative to Ler-0 wild type (Figures 2A, B, E, F; Figures S5, S6, S7 and S8, black arrows). For *SCL6(IV)/*AT4G00150 and *TOE2/*AT5G60120, there were 1 to 2.5- fold increases relative to wild type (Figures 2C and D; Figures S9 and S10). In the *dcl1-7* mutant, the relative expression levels of antisense transcripts for five genes were decreased by 20–40%, including *APS1*, *MYB12*, *SCL6(IV)*, *DCL1/*AT1G01040, and *SPL10/*AT1G27370 (Figures 3, S5, S6, S9, S11, S12). The *hyl1-2* mutant had decreased antisense transcript expression by 20–50% for *APS1*, *MYB12*, *SCL6(IV)* and *TOE2* (Figures 4, S5, S6, S9, S10). Conversely, *ARF17/*AT1G77850 and *MET2/*AT4G14140 antisense transcript expression levels were up-regulated in *hyl1-2* (Table S7, Figure S13), and there was a more complex pattern of expression for *TCP4/*AT3G15030 antisense transcripts that were elevated in the upstream region while decreased in the downstream region in *hyl1-2* (Figure S14).

**Figure 5 pgen-1000457-g005:**
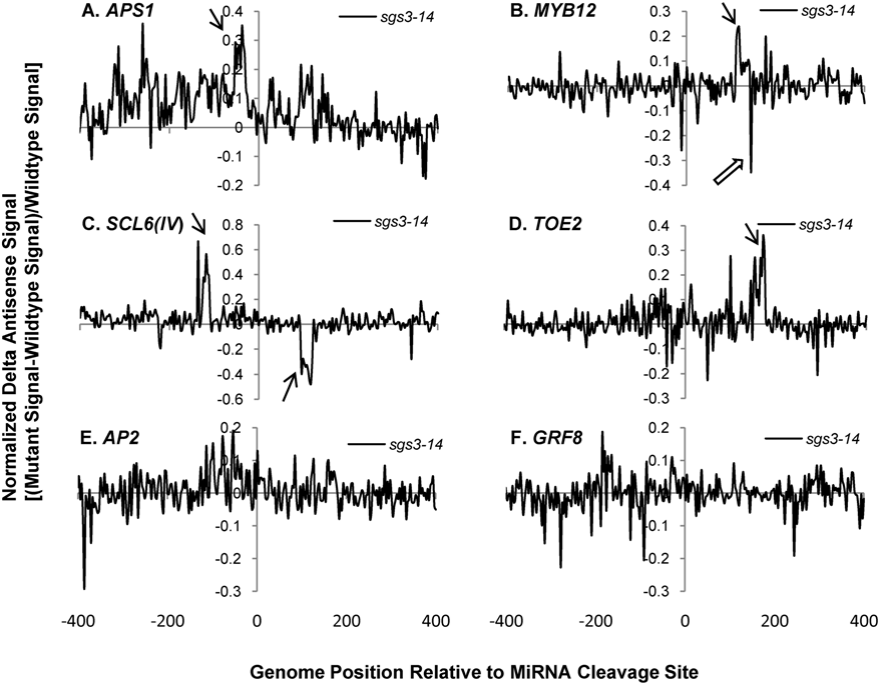
Normalized delta signals for antisense transcripts of selected validated miRNA targets showing differences between *sgs3-14* versus wild type Col-0. Refer to Figure 2 for details of legend. The open arrow in panel B points at the decreased antisense signal adjacent to the increased antisense signal for *MYB12* in *sgs3-14* mutants.

**Figure 6:**
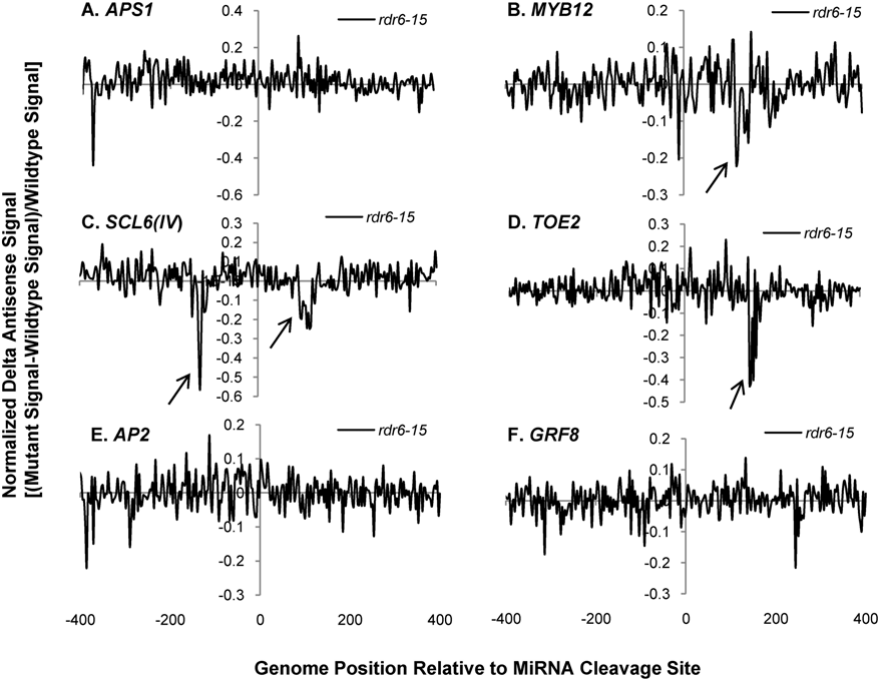
Normalized delta signals for antisense transcripts of selected validated miRNA targets showing differences between *rdr6-15* versus wild type Col-0. Refer to Figure 2 for details of legend.

Another striking observation was seen in the *sgs3-14* mutant: *APS1*, *MYB12*, *TOE2*, *DCL1*, *SPL10*, and *TCP4* had increased expression of antisense transcripts (Figures 5A, B, D; S5, S6, S11 S12, S14). For *MYB12*, *SCL6(IV)* and *TCP4*, there were some antisense transcripts with complex changes corresponding to increases as well as decreases (Figures 5B, C; S6, S9, S14). In the *rdr6-15* mutant, *MYB12*, *SCL6(IV)*, *TOE2*, and *TCP4* antisense transcript expression was down-regulated, while there was an increase of *UBC24/*AT2G33770 antisense transcripts (Figures 6B–D; S6, S9, S10, S14, S15). Taken together, around 80% of the sixteen validated miRNA targets were elevated in the *hen1-1* mutants for the antisense transcript expression, whereas about a quarter to one third of these 16 targets were affected in one of the other four smRNA pathway mutants, including *dcl1-7*, *hyl1-2*, *rdr6-15* or *sgs3-14*. *MYB12* and *SCL6(IV)* were affected by all five mutants in that there was elevated antisense transcript expression in *hen1-1*, complex up and down signal levels in *sgs3-14*, and decreased expression in *dcl1-7*, *hyl1-2* and *rdr6-15*. Because the antisense transcript topologies were replicated precisely (i.e. in the same probe sets) in completely different sets of experiments with different control genotypes Landsberg *erecta* and Columbia (Ler-0, Col-0), we conclude that despite their low abundance relative to sense transcripts, the antisense transcripts mapping near to the miRNA binding sites of target genes are highly reproducible.

Some general features characterize the identified antisense transcripts: (1) the expression peaks appeared to be concordant with sense transcripts. For example, comparison between the wild type sense and antisense strand raw signals for *AP2* and *SPL10* showed that these genes with introns in the probe set had no antisense transcripts in the sense intronic region (Figures S2A, S4E, S2F, and Table S6). This suggested the antisense transcripts associated with miRNA targets were generated from the mature mRNA transcripts. Supporting evidence comes from *APS1*, *AP2* and *SPL10* which also had concordant changes in antisense signals to sense signals in smRNA pathway mutants (Figures S5, S7, S12). (2) The effect on antisense transcript abundance by smRNA pathway mutants did not strictly correlate with that of sense transcripts expression except for a few cases in *hen1-1* and *sgs3-14*. For instance, elevated expression of *DCL1* antisense transcripts in *hen1-1* and *sgs3-14* mutants was not correlated to that of sense transcripts which were unchanged in these two mutants (Figure S11). A similar situation was seen for *MET2*, where the antisense transcripts of *MET2* were increased in the *hyl1-2* mutant. Nevertheless, its sense transcript abundances were unchanged in the corresponding mutant (Figure S13). In some other cases, the antisense transcripts had reciprocal expression patterns compared to their cognate sense transcripts, for example, *MYB12* in *hyl1-2* and *sgs3-14*, *SCL6(IV)* in *dcl1-7*, *sgs3-14* and *rdr6-15*, and *TOE2* in *hyl1-2* and *rdr6-15* (Figures S6, S9, S10). This suggested a possible regulatory function of antisense transcripts on their coordinate sense transcripts. For *hen1-1* mutants, most antisense transcripts of validated miRNA targets were elevated along with their sense transcripts. We interpret the increased antisense transcripts as an indirect consequence of the increased stability of their sense transcripts due to the loss of function of HEN1 in the mutant, because for some targets, such as *DCL1* and *MET2*, the antisense transcripts were up-regulated whereas the levels of their sense transcripts did not change (Figures S11, S13). For *CC-NBS-LRR/*AT5G43740, the observed increases in antisense transcript abundance were accompanied by a concordant decrease of its cognate sense transcript expression in *hen1-1* (Figure S16). In general, these observations support the notion that the increased antisense transcripts associated with miRNA targets are due to the loss of HEN1 function, presumably due to the loss of the 2′-methylated hydroxyl group on the 3′ end of smRNAs in the *hen1-1* mutant [22]. (3) In *sgs3-14*, adjacent probes for *MYB12* and *TCP4* reported signals of widely differing amplitudes, where a few probes showed high signals (black arrows in Figures 5B, S6 and S14) and nearby probes recorded decreased signals relative to wild type (open arrows in Figures 5B, S6 and S14). The variable effects of *sgs3-14* on transcript topology suggested a dynamic process affecting antisense transcript stability, which may also explain the complex expression pattern for the antisense transcripts with *SCL6(IV)* and *TCP4* in *dcl1-7* or *hyl1-2* (Figures 3 and S14). We propose this phenomenon seen with the high resolution microarray is evidence of transitive mechanisms in action, e.g. rapid smRNA production by the cleavage of antisense and/or sense transcripts detected as fluctuating microarray signals.

### Validation and Extension of Microarray Data by Semi-Quantitative Strand-Specific Reverse Transcription PCR

**Figure 7:**
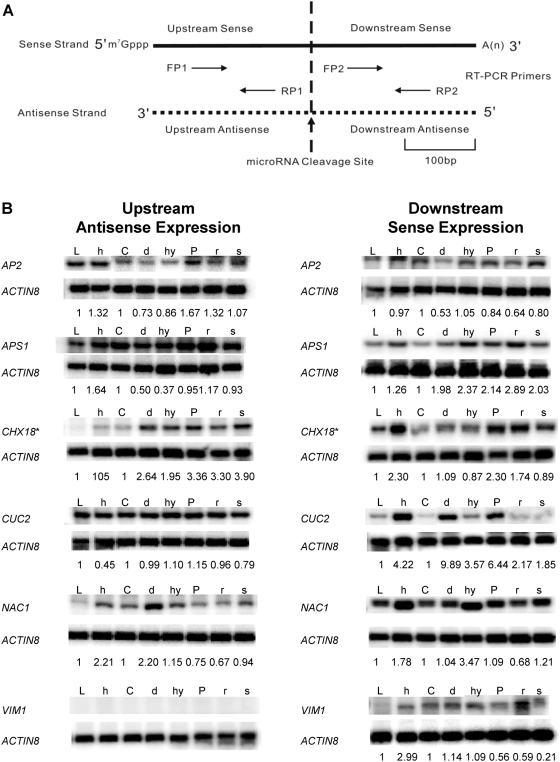
qRT-PCR for sense and antisense expression of selected miRNA targets. (A) Cartoon showing experimental design. For each selected miRNA target, two pairs of primers are designed, one pair located upstream of the miRNA cleavage site (dashed vertical line) labeled as FP1 and RP1 (forward primer1 and reverse primer1), and another pair located downstream of the miRNA cleavage site labeled as FP2 and RP2 (forward primer2 and reverse primer2). Regions queried (upstream or downstream) are defined according to their positions relative to the miRNA cleavage site. Approximate scale of average-sized PCR products (∼100 b.p.) is indicated. (B) qRT-PCR results for validated miRNA targets *AP2*/AT4G36920, *APS1*/AT3G22890, *CHX18*/AT5G41610, *CUC2*/AT5G53950, *NAC1*/AT1G56010 and a paralogous non-target *VIM1*/At1g57820. On the right panel “Downstream Sense Expression”, the primer RP2 was used in the reverse transcription and primers FP2+RP2 were used in the following PCR reaction. On the left panel “Upstream antisense expression”, the primer FP1 was used in the reverse transcription and primers FP1+RP1 were used in the following PCR reaction. *ACTIN8* primer pairs for sense strand expression were included in each qRT-PCR reaction as a duplexed semi-quantitative internal control. The relative expression value of each qRT-PCR band normalized to its *ACTIN8* signal is indicated below each lane. No band was detected when reverse transcriptase was omitted from the reverse transcription reaction in negative controls (data not shown). L: wild type Ler-0; h: *hen1-1*; C: wild type Col-0; d: *dcl1-7*; hy: *hyl1-2*; P: a P1/HC-Pro over-expressing line; r: *rdr6-15*; s: *sgs3-14*. Each panel is a representation of at least three independent replicates from each of two biological samples that gave similar results. Asterisk (*) in the panel for *CHX18* denotes the region upstream or downstream of the miR856 cleavage site on *CHX18* mRNA.

qRT-PCR was employed for select miRNA targets on the microarray as well as for other miRNA target genes. qRT-PCR primers were designed from ∼200 n.t. range 5′ upstream and 3′ downstream of the miRNA cleavage sites (Figure 7A) for *AP2*, *APS1*, *CATION/H+ EXCHANGER 18* (*ATCHX18/*AT5G41610)(miR856 cleavage site), *CUC2/*AT5G53950, *NAC1/*AT1G56010 and a negative control gene *VARIANT IN METHYLATION 1 (VIM1)/*AT1G57820 previously shown not cleaved by miR164 [27]. The results of qRT-PCR for sense strands were generally consistent with previous [19] and our custom tiling microarray results (Figure S2). *AP2* sense transcript expression was unchanged in *hen1-1*, *hyl1-2* and *sgs3-14*, whereas it was decreased in *dcl1-7* and *rdr6-15* (Figure 7B right panel “Downstream sense expression”). Also in agreement with the microarray data was the finding that *AP2* antisense transcripts were increased by ∼30% in *hen1-1* mutants, and decreased in *dcl1-7*. We also examined the effect of a RNA silencing suppressor protein P1/HC-Pro from Turnip mosaic virus which binds to the miRNA/miRNA* duplex and probably inhibits the 3′-terminal methylation of smRNAs [34]. We found that *AP2* antisense transcripts were up-regulated in a *P1/HC-Pro* over-expressing line. A slightly higher expression was observed by qRT-PCR for antisense transcripts in the *rdr6-15* mutant than by microarray analysis (compare Figure 6E with Figure 7B). *APS1* sense transcripts were increased in all mutants, supporting the microarray results for *hen1-1* and *sgs3-14*, but in contrast to those for *dcl1-7*, *hyl1-2*, and *rdr6-15* (Figure S5). The differences observed might be due to sensitivity limitations (note the low signal to noise ratios for Figures 2–6 in some cases) or amplification differences inherent to the two methods. *APS1* antisense transcripts were upregulated in *hen1-1*, down-regulated in *dcl1-7* and *hyl1-2*, which was congruent with tiling array results. *ATCHX18* is a member of putative Na+/H+ antiporter family targeted by miR856 and miR780. The expression level of the downstream sense region for the miR856 target site was increased in *hen1-1*, P1/HC-Pro lines, and *rdr6-15*, whereas corresponding upstream antisense transcripts were elevated in all mutants (Figure 7B). Interestingly, a natural antisense transcript (AT5G41612; TAIR Release 8) overlaps with *ATCHX18* and might be queried in the qRT-PCR assay, despite the primers being over 1 kb distal to the annotated natural antisense transcript. *CUP-SHAPED COTYLEDON 2 (CUC2)* and *NAC1* are members of NAC domain-containing transcription factors and are validated targets of miR164. qRT-PCR data showed that *CUC2* sense transcripts were up-regulated in all mutants, whereas the levels of its antisense transcripts were unchanged in most mutants except for a decrease in *hen1-1*. *NAC1* had more sense transcript expression in *hen1-1* and *hyl1-2* and less expression in *rdr6-15*. For *NAC1* antisense transcripts, expression was elevated in *hen1-1* and *dcl1-7*, but decreased in *rdr6-15* (Figure 7B). *VIM1* encodes a SRA (SET- and RING-associated) domain methylcytosine-binding protein, and it has been shown to have a cryptic miR164 binding site that fails to generate a cleavage product as probed by 5′-RACE [27]. Thus, it was selected as a reference control for the qRT-PCR assays. *VIM1* locus clearly showed some altered sense transcripts in the smRNA pathway mutants, however, as hypothesized, no antisense transcripts were detected under experimental conditions (Figure 7B).

**Figure 8:**
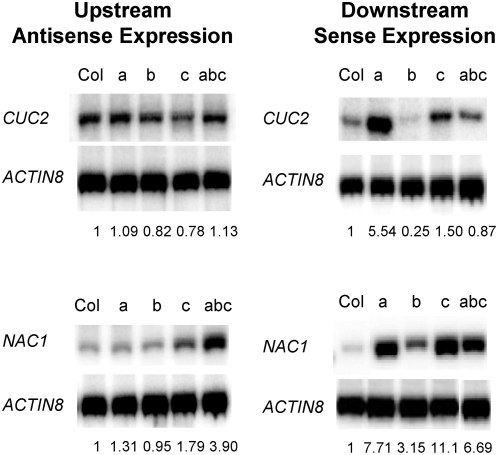
qRT-PCR for selected miRNA targets in different *mir164* knockout mutants. *CUC2/AT5G53950* and *NAC1/AT1G56010* sense and antisense transcript expression was analyzed in the RNA extracted from the aerial parts of whole plants of the following genotypes: Col: wild type Columbia-0; a: *mir164a-4*; b: *mir164b-1*; c: *mir164c-2*; abc: *mir164a-4 b-1 c-1* [35]. See Figure 7 for details of legends.

In order to test the functional significance of *MIR164* expression on transcripts of *CUC2* and *NAC1*, their sense and antisense transcript levels were assayed in *mir164a-4*, *mir164b-1*, *mir164c-2* single mutants and *mir164a-4 b-1 c-1* triple knockout mutants [35]. As expected, *CUC2* sense transcripts accumulated in the *mir164a-4* and *mir164c-2* mutants (Figure 8 right panel), but the antisense transcripts of *CUC2* were unchanged in these knockout mutants except for a slight decrease in the *mir164c-2* mutant (Figure 8 left panel). *NAC1* sense transcript levels were elevated in all the knockout mutants and its antisense transcripts also increased in *mir164a-4*, *mir164c-2* and *mir164a-4 b-1 c-1* mutants (Figure 8). These results suggest that miR164 is probably not a primer for the observed antisense transcription, as previously speculated based on the function of HEN1 as a methyltransferase [36]. Northern blot for miR164 expression from inflorescence samples of these mutants showed that even in *mir164a-4 b-1 c-1* triple mutants, miR164 expression was not completely abolished with ∼20% detectable expression level comparing to that of wild type [35]. The expression of a distinct miR164 species of 24-n.t. in length was generally unchanged in all these *mir164* single and triple mutants [35]. These results imply that there should be more direct determinants regulating the abundance of miRNA target-associated antisense transcripts other than miRNAs themselves.

### MiRNA Targets and *MIRNA* Genes Are Hot Spots for Generating smRNAs

**Figure 9:**
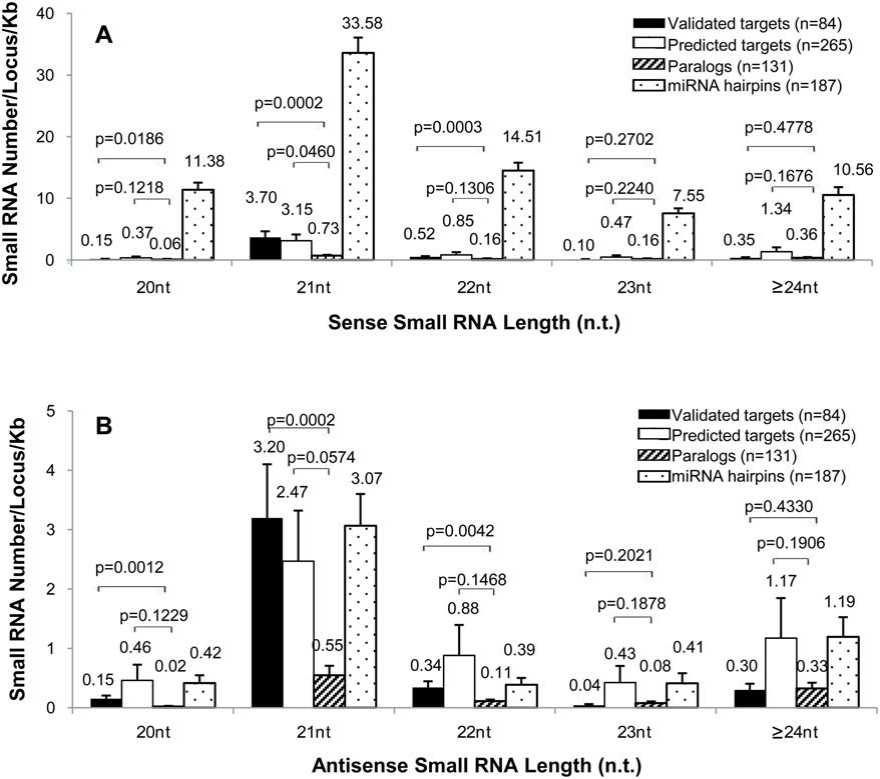
Normalized abundance of unique smRNAs from multiple deep sequencing datasets with perfect matches to miRNA-associated gene sets. (A) Number of unique smRNAs mapping to the sense strand of validated or predicted miRNA target genes, paralogous non-targets and *MIRNA* hairpins. (B) Number of unique smRNAs mapping to the antisense strand of validated and predicted miRNA targets, paralogous non-targets and *MIRNA* hairpins. smRNA sequences were obtained from published data [20],[28],[37],[38] and miRNA hairpin sequences were queried from the miRBase database (http://microrna.sanger.ac.uk/) [76]. The number of unique smRNAs were found by BLAST against the cDNA sequences or miRNA hairpins and then normalized by the length of each individual matching gene (see “Material and Methods” for details). The average number for each set of genes is presented here. Standard error bars are indicated in the plot. *P* values of Student's t-test (one-sided, equal variance assumed) are shown above the brackets between different groups.

The availability of deep sequencing datasets for smRNAs [20],[28],[37],[38] affords the means to correlate antisense transcript abundances with their presumptive DCL products and gain insight into the causal relationships of antisense transcripts and smRNAs. We mined the unique smRNAs having only one locus in the *A. thaliana* genome that matched perfectly to the sense or antisense strand of test sets of miRNA-associated genes (Table S8). Figure 9 shows the average number smRNAs of different size classes normalized for gene length in validated or predicted miRNA targets, paralogous non-targets, and *MIRNA* genes. In the categories of 20–22 n.t. smRNAs, validated miRNA targets had significantly more smRNAs matching to the sense strand compared to paralogs (Figure 9A, *P*<0.05, one-sided Student's t-test, equal variance model), especially in the size class of 21 n.t. Predicted miRNA targets also generated abundant smRNAs, in which 20, 22, 23, and ≥24 n.t. groups gave higher numbers of smRNAs from the sense strand when compared with validated miRNA target genes. The 21 n.t. predicted target-originated sense smRNAs were significantly more abundant than those from paralogs (Figure 9A). For reference, the number of sense strand smRNAs generated from 187 miRNA hairpins (miRBase, microrna.sanger.ac.uk) was also calculated. MiRNA hairpins produced predominantly 20–22 n.t. smRNAs, which is well known as due to the processing of miRNA hairpin precursors to generate mature miRNAs and miRNA* by DCL1 and/or DCL4 [28]. MiRNA hairpins also produced 23–24 n.t. and longer smRNAs, consistent with a report on functional 23 to 25 n.t.-long miRNAs generated by DCL3 [39], indicating the overlapping functions of different DCLs on the processing of miRNA hairpin precursors. The antisense strand of miRNA targets produced smRNAs to a similar extent as those from the sense strand compared to paralogs (Figure 9B). Validated miRNA targets had significantly more 20–22 n.t. smRNAs than paralogs (*P*<0.05, one-sided Student's t-test, equal variance model). The 21 n.t. sense and antisense smRNAs were the main class of smRNAs generated from validated and predicted miRNA targets, suggesting they are mechanistically linked to the RNA silencing pathway through DCL1. Remarkably, *MIRNA* hairpins generated antisense smRNAs as well, in which 21 n.t. antisense smRNA were also the major class (Figure 9B). Table 2 summarizes the known cases of miRNA targets and their *MIRNA* genes that generated antisense smRNAs, ranked according to abundances of antisense smRNAs and grouped into MIRNA gene families. It is interesting that several of the transitive *MIRNA* genes correlate with top-ranking miRNA targets, for example *ATCHX18* and *MIR780*, *AGO1* and *MIR168a*, *SCL* family and *MIR171c*, *SAMT* and *MIR163*, *AP2* and *TOE2* with *MIR172*, and the *SPL* family with *MIR156* (Table 2). Careful analysis of the location for these sense and antisense smRNAs on the miRNA hairpins showed that about 30% of unique sense smRNAs overlap with mature miRNA sites, whereas another 28% overlap with the miRNA* sites by at least 16 n.t. (Figure S17). For the unique antisense smRNAs on the miRNA hairpins, about 14% overlap with the locus of the mature miRNA on the sense strand, whereas 27% of them overlap with the miRNA* sites Interestingly, several antisense 24 n.t. smRNAs were found to be in phase with the middle of the mature miR783 or miR854b* site on their individual hairpins (Figure S18). We propose this is evidence for the miRNA hairpin processing via the RNA silencing pathway in which the miRNA* or miRNA may be programmed into a RISC that triggers cleavage [29] and/or antisense transcription and subsequent dicing on their primary transcripts, in these cases presumably by DCL3.

**Table 2:** Validated miRNA targets and their associated *MIRNA* genes generating unique antisense (α-) smRNAs^a^.

Validated Target	Gene Name	Normalized α-smRNA reads^b^	*MIRNA* Gene	Normalized α-smRNA reads^b^	Reference
AT3G23690	*bHLH*	69.86	*MIR393a, b*	0	[15],[23]
AT1G12820	*AFB3*	34.08	*MIR393a, b*		[15],[23]
AT3G26810	*AFB2*	21.23	*MIR393a, b*		[15],[16],[23]
AT3G62980	*TIR1*	7.26	*MIR393a, b*		[15],[16]
AT1G27340	*F-box*	2.16	*MIR393a, b*		this work
AT5G41610	*ATCHX18*	60.61	*MIR780, MIR856*	5.75, 0	[23]
AT5G43740	*CC-NBS-LRR*	54.61	*MIR472*	0	[23]; this work
AT1G48410	*AGO1*	17.67	*MIR168a, b*	7.25, 0	[15],^20^,^23^,^77^
AT4G14140	*MET2*	12.72	*MIR773*	0	this work
AT4G00150	*SCL6*	12.45	*MIR171c*	8.62	this work
AT2G45160	*SCL*	10.92	*MIR171c*		this work
AT3G60630	*SCL*	8.91	*MIR171c*		this work
AT1G66720	*SAMT*	11.65	*MIR163*	33.23	[78]; this work
AT3G44860	*FAMT*	4.05	*MIR163*		[78]; this work
AT4G36920	*AP2*	9.74	*MIR172a, b, c, d, e*	19.61, 10.53, 7.52, 8.06, 32	[20]; this work
AT5G60120	*TOE2*	3.31	*MIR172a, b, c, d, e*		[20]; this work
AT1G53230	*TCP3*	7.59	*MIR319a*	11.36	this work
AT4G18390	*TCP*	4.88	*MIR319a*		this work
AT3G15030	*TCP4*	2.48	*MIR319a*		this work
AT1G66370	*MYB113*	5.39	*MIR828*	0	this work
AT1G06580	*PPR*	4.38	*MIR161*	40.46	[15]
AT5G43270	*SPL2*	4.21	*MIR156b, d, e, g*	5.46, 25.42, 9.35, 9.71	this work
AT2G33810	*SPL3*	3.05	*MIR156b, d, e, g*		this work
AT1G27370	*SPL10*	2.20	*MIR156b, d, e, g*		[16]; this work
AT3G57230	*AGL16*	4.06	*MIR824*	1.45	this work
AT3G19890	*F-box*	4.05	*MIR774*	10.2	this work
AT2G33770	*UBC24*	3.90	*MIR399a∼f*	0	this work
AT1G30330	*ARF6*	3.88	*MIR167a, c, d*	7.25, 6.25, 7.96	this work
AT5G37020	*ARF8*	3.16	*MIR167a, c, d*		this work
AT1G02860	*NLA*	3.29	*MIR827*	0	this work
AT1G01040	*DCL1*	3.20	*MIR162a, b*	7.14, 27.03	this work
AT5G07680	*ATNAC4*	2.93	*MIR164a, b, c*	0	this work
AT1G56010	*NAC1*	2.87	*MIR164a, b, c*		this work
AT3G08500	*MYB83*	2.92	*MIR858*	5.35	this work
AT1G08830	*CSD1*	2.29	*MIR398a, b, c*	0	this work
AT1G52150	*ATHB15*	1.99	*MIR166e*	6.99	this work
AT1G30490	*PHV*	1.70	*MIR166e*		this work
AT2G34710	*PHB*	1.31	*MIR166e*		this work
AT1G77850	*ARF17*	1.93	*MIR160a, b, c*	0	this work
AT2G28350	*ARF10*	1.77	*MIR160a, b, c*		this work
AT5G06100	*MYB33*	1.88	*MIR159a*	5.43	this work
AT3G11440	*MYB65*	1.49	*MIR159a*		this work
AT1G31280	*AGO2*	1.79	*MIR403*	0	this work
AT1G17590	*NF-YA8*	1.64	*MIR169a, i, j*	13.27, 19.42, 13.57	this work
AT2G36400	*AtGRF3*	1.24	*MIR396a*	19.87	this work

a smRNA sequences were collected from published data [20],[28],[37],[38].

b The number of antisense smRNAs with perfect matches to the cDNA for each validated miRNA target and each miRNA hairpin was scored and then divided by the length of each gene or hairpin individually (antisense smRNA number/kb). *TAS* genes targeted by miR173, miR390, and miR828 were excluded from this analysis.

**Figure 10:**
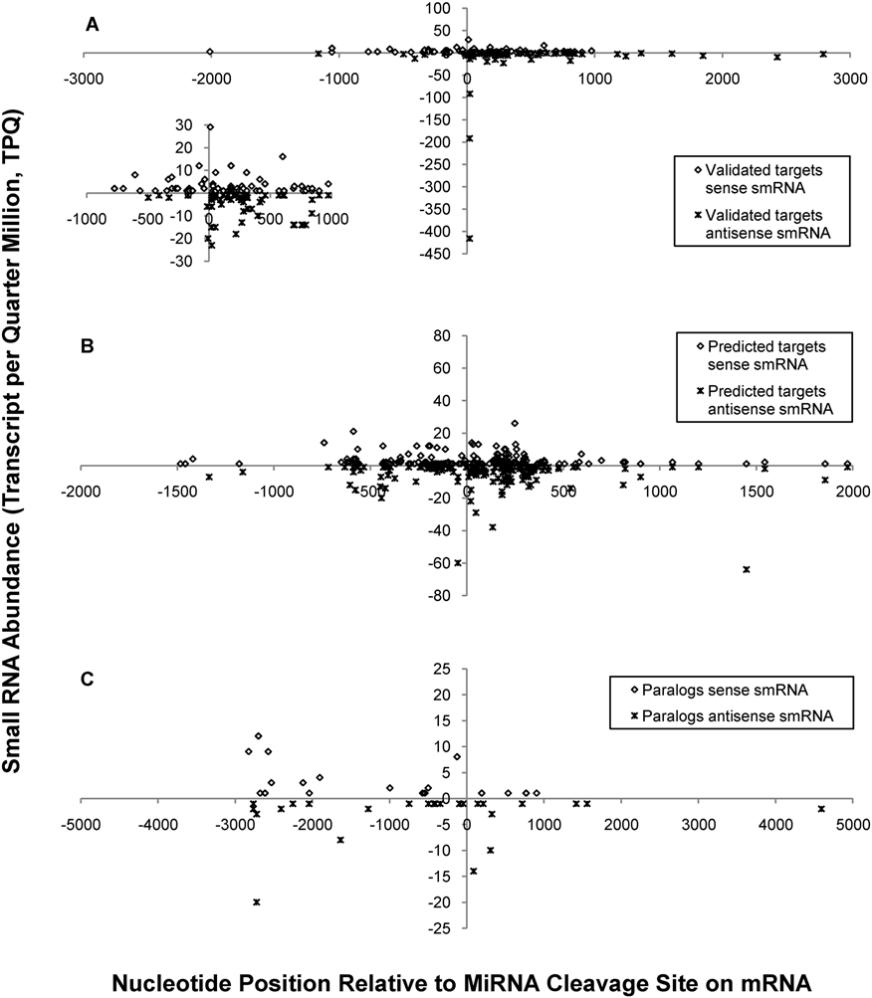
Abundance and positions of unique MPSS smRNA signatures with perfect matches to miRNA targets and paralogous non-targets. (A) Validated miRNA targets; (B) Predicted miRNA targets; (C) paralogous non-targets. MPSS smRNA signatures were obtained from the MPSS Plus Database (http://mpss.udel.edu/at) and searched against *A. thaliana* cDNA sequences to find the unique matches by BLAST (see “Material and Methods” for details). The abundance of unique signatures (transcripts per quarter million) is plotted as a function of the position of signatures relative to the miRNA target sites for validated and predicted miRNA targets, or to pseudo binding sites for paralogous non-targets. Sense smRNAs are plotted on the positive-valued ordinate of each panel, while antisense smRNAs are on the negative-valued ordinate. The inset in panel A shows expanded ordinate scale for the distribution of smRNAs spanning 1,000 n.t. upstream and downstream of miRNA cleavage sites for validated targets.

We further investigated the topology of antisense transcription manifested in smRNAs by plotting the abundance of unique smRNAs (extracted from the MPSS Plus database) as a function of the distance between the smRNA loci and the miRNA target sites for validated miRNA targets, predicted targets and paralogous non-targets (Figure 10). Validated targets had sense and antisense smRNAs clustered around 1000 n.t. upstream and downstream of the miRNA cleavage sites, with a few cases of hits >2000 n.t. upstream and 3000 n.t. downstream of the cleavage sites (Figure 10A). The numbers of sense: antisense smRNA signatures associated with validated targets were about the same (70: 62; Table S9). However, the topology of these smRNA signatures showed that the numbers of sense and antisense smRNA signatures downstream of miRNA cleavage sites were greater than those upstream (22 up: 48 down and 7 up: 55 down for sense and antisense smRNA signatures, respectively; Table S9 and Figure 10A inset). Antisense smRNAs were significantly more abundant than the sense smRNA signatures even when the two most abundant antisense smRNA signatures were removed (transcripts per quarter million = 416 and 192 corresponding to *NF-YA8/*AT1G17590 [miR169 target]; *ATHB15/*AT1G52150 [miR166 target], respectively; *P*<0.05, one-sided Student's t-test, equal variance model). This same phenomenon was observed in predicted miRNA targets as well, with significantly higher abundances for antisense smRNA signatures than sense smRNA ones (*P*<0.05, one-sided Student's t-test, equal variance model; Table S9, Figure 10B). There were also more antisense smRNA signatures located downstream of the predicted miRNA cleavage sites than upstream antisense ones (50 up: 113 down, respectively). Paralog genes showed no significant correlation (Table S9, Figure 10C). These results indicate that generally more smRNA signatures were generated towards the 3′ end of miRNA target transcripts, presumably from the downstream region of the miRNA cleavage sites on the antisense strand. These data fit with the observation that uncapped transcripts are more susceptible to RNA silencing pathways, which lead to the production of sense and antisense smRNAs [33].

## Discussion

Production of antisense transcripts is a pervasive but poorly understood phenomenon and it has been scrutinized as a potential artifact in transcriptome experiments [40],[41]. By combining different techniques and analyses, including custom high resolution tiling microarrays, qRT-PCR and computational analysis of whole genome tiling array and deep-sequencing smRNA data, we show that there are significantly larger numbers and abundances of antisense transcripts and smRNAs associated with validated miRNA targets than with non-target paralogs (Figures 1, 7, 9, 10). *MIRNA* genes also produce substantial and significant numbers of antisense smRNAs (Figure 9; Table 2), implicating the involvement of antisense transcription in miRNA hairpin processing. The miRNA target-associated antisense transcripts were reproducible in abundance and topology (Figures S4, S5, S6, S7, S8, S9, S10, S11, S12, S13, S14, S15, S16). Unambiguous antisense transcripts to several miRNA target genes depended on DCL1 and HYL1 (Figures 3 and 4) and RDR6 (Figure 6), whereas they inversely relied on two heretofore unrelated components, HEN1 and SGS3 (Figures 2 and 5). All these findings are compelling evidence that antisense transcription is biologically significant, at least in the class associated with miRNA targets and, by inference, associated with *MIRNA* genes. The transitive process of antisense transcription and production of secondary smRNAs may be an important aspect of miRNA target and *MIRNA* gene expression. Supporting evidence can be found in the highly-abundant RDR6-dependent antisense smRNAs which are located exactly downstream of the miRNA cleavage sites of *AGO1*, *AFB3*, and *TIR1* target transcripts [15],[16],[20],[23]. However, the molecular mechanisms triggering production of these specific antisense transcripts await further elucidation.

### Mechanisms of Production of MiRNA Target-Associated Antisense Transcripts

To date, two models have been proposed for post-transcriptional gene silencing which can be applied to the question of miRNA-associated transitivity in terms of generation of antisense transcripts and secondary smRNAs: 1) RDRs may use rare primary siRNAs to “prime” (in the formal sense) dsRNA using the target mRNA as template, i.e. extend the dsRNA into the 5′ (upstream) end of the sense transcript [42],[43],[44]. 2) Copy RNA synthesis may occur by un-primed initiation, supported by the evidence that siRNAs spread both 5′ and 3′ along the target relative to the trigger in plants and Neurospora [45],[46]. There is biochemical evidence for both pathways [43],[45] and they probably overlap at some key point(s) in the pathways. The situation is confounded by the issue of causality: the generation of secondary smRNAs could be the consequence of, or the source of, antisense transcripts. There are several unanswered questions that impact the origin of miRNA-associated antisense transcripts and secondary smRNAs: 1) Is miRNA or smRNA required as primer? 2) What are the sources of template that serve as triggers for these antisense transcripts? 3) Is there any specificity determinant involved in the process?

Concerning the requirement of miRNA as primer in the miRNA target-associated antisense transcription, Ronemus *et al.* [16] have suggested that transcription activity in the complementary region to 5′ upstream targeted sequences on miRNA targets might correlate with those miRNAs which have 3′ ends that match perfectly to their targets. However, we observed strong transcription signals and upstream smRNAs in many targets regulated by miRNAs that have substantial 3′ mismatches (e.g. Figure S1; data not shown). HEN1 is a methyltransferase involved in the methylation of 2′-OH on the 3′ end of miRNAs and siRNAs [22],[36]. The methylated 2′-OH is postulated to protect the 3′ end of smRNAs from uridylation and presumably from antisense transcription of template strands that share high homology with miRNAs or siRNAs [22]. Loss of HEN1 function alters miRNA abundances and exposes the free 3′ end of smRNAs, which might serve as triggers via priming *per se* or otherwise in the generation of antisense transcripts. In the *hen1-1* mutant, the expression of antisense transcripts for 80% of examined miRNA targets on our custom tiling microarray increased substantially relative to wild type (Figure 2; Table S7). This is consistent with an indirect (non-priming) trigger mechanism when taken in light of the abundance of secondary smRNAs mapping downstream of cleavage sites (Figure 10A) and assuming that antisense transcripts are causal to smRNA production. We hypothesize there should be homeostasis between an antisense transcription pathway and the degradation of smRNAs by a family of exoribonucleases encoded by the *SMALL RNA DEGRADING NUCLEASE* (*SDN*) genes [47], raising the issue of the steady state levels of “functional” miRNAs and siRNAs in *hen1-1* that could impact the hypothesized trigger for antisense transcription. Another indirect evidence for dispensability of miRNAs as primers is that RDR6 possesses primer-independent RNA polymerase activity on single-stranded RNAs no matter the substrate has a cap or poly(A) tail [48]. This fact indicates that at least in RDR6-dependent antisense transcription, priming activity by miRNAs is not needed and indeed most of our data do not support a requirement for RDR6 in antisense transcription of miRNA targets (Figures 6, 7; Table S7).

Regarding the source of templates in miRNA target and *MIRNA* gene-associated antisense transcription, the 5′ and 3′ cleavage fragments of miRNA targets and pri-miRNAs targeted by RISCs could serve as a supply. It is reported that transcripts without a cap or a poly(A) tail are preferentially directed to the RNA silencing pathway and secondary siRNAs could be generated from these “aberrant” RNA transcripts [33],[49],[50],[51] by antisense transcription. Similar to the catabolism of smRNAs, there are known degradation pathways (containing 3′ to 5′ or 5′ to 3′ exoribonucleases [52],[53]) for the mRNA cleavage fragments that compete with RNA silencing pathways in Arabidopsis [54]. In human cells, the addition of a 3′ terminal oligo U-tract on mRNAs or mRNA fragments can promote decapping and stabilization of the 3′ end of the RNA by binding the Lsm1-7 complex that ensures 5′-directional degradation [55]. This implies the 3′ end of the 5′ fragment of miRNA target transcripts in Arabidopsis could be stabilized by a similar mechanism and would have a longer half life than its 5′ end, thus increasing the probability for it to serve as a template for RNA silencing. For the 3′ endonucleolytic fragment of miRNA targets, the lack of a 5′ cap could facilitate its entry into RNA silencing pathways in competition with the surveillance of the EXORIBONUCLEASE 4/ETHYLENE INSENSITIVE 5 (XRN4/EIN5) and/or ABA-HYPERSENSITIVE-1/CAP BINDING PROTEIN80 (ABH1/CBP80) [33].

Our observation of SGS3-dependent accumulation of sense and antisense transcripts for several miRNA targets that produce siRNAs (Figures 5 and 7; Figures S5, S10, S11, S14) supports the notion that SGS3 could be a determinant in the production of miRNA target-associated antisense transcription. SGS3 is predicted to encode a coiled-coil RNA binding protein with a novel XS domain [56],[57]. SGS3 functions as a key component of the unprimed post-transcriptional transgene- and virus-induced gene silencing pathway [24],[58]. It is also required for vegetative phase change mediated by targets of miR156 that produce antisense transcripts [21]. Many of the same genes are up-regulated in *sgs3*, *asymmetric leaves1 (as1)*, and *ago7/zippy* mutants [14],[59] and we postulate that these altered genes may produce antisense transcripts that are important for gene regulation. Yoshikawa *et al.* [60] reported that SGS3, RDR6 and DCL4 work sequentially to generate the 21 n.t. species of smRNAs from the 3′ cleavage fragment of *TAS1/2*, while the 24 n.t. smRNAs are dependent on DCL3. SGS3 stabilizes the 3′ cleavage fragments of *TAS1a* and *TAS2* transcripts [60], but it is unknown why the 5′ cleavage fragments of *TAS1a* and *TAS2* can accumulate in *sgs3-11* and generate 24 n.t. smRNAs. We speculate that SGS3 involvement in the production of miRNA target-associated antisense transcripts might be uncoupled from RDR6 or require other RDRs, for example RDR1 or RDR2. SGS3 might be a transporter/stabilizer of cleaved products of miRNA targets, analogous to the LSm1-7 complex in humans. It could bind the single-stranded cleavage fragments of miRNA targets and promote their 5′ to 3′ degradation. Loss of function for SGS3 would channel these cleavage products into the RNA silencing pathway mediated by RDR(s) as shown for RDR6-dependent *TAS1/2/3* processing. This pathway for metabolism of unstable transcripts would be in competition with the mRNA degradation pathways, including the 3′ to 5′ exosome or the 5′ to 3′ exoribonucleases [52],[53].

### Possible Biological Significance of MiRNA Target-Associated Antisense Transcripts

The production of antisense transcripts and antisense smRNAs from the miRNA targets probably induces a series of subsequent reactions in vivo. Antisense transcripts are prerequisites for formation of long dsRNA duplexes which may function in post-transcriptional gene silencing as hypothesized for natural antisense transcripts [61]. This could result in the generation of secondary smRNAs and probable down-regulation of transcripts with little homology to the primary smRNAs. This action would likely be restricted to some specific cell types or some extreme physiological conditions such that it would not affect the normal biological functions of the cognate genes in vivo. Our finding that not every miRNA target gene generates antisense transcripts or smRNAs is in line with this notion. Another aspect is that the antisense smRNAs and antisense transcripts can function in transcriptional gene silencing by DNA or chromatin modifications. Recent results show that human genes are regulated transcriptionally by promoter-associated and terminator-associated antisense RNAs that are targets of the exosome [62],[63],[64],[65]. Other examples are the *p21* and *E-cadherin* genes that have antisense transcripts which produce smRNAs that drive transcriptional gene silencing of the cognate genes [66].

Our findings suggest the existence of a novel antisense pathway generating RNA transcripts complimentary to the sense strand of miRNA target mRNAs. However, we believe such transitivity is under stringent control for the majority of non-*TAS* miRNA targets, as evidenced by the elucidation of a downstream antisense transcription pathway for some miRNA targets that mimics ta-siRNA pathways (Figure S1) [15]. Because miRNAs are under strong selection pressure for their target mRNAs and act dominantly, their cell-specific expression must be tightly regulated. Therefore, transitivity may be under negative selective pressure because extensive amplification would compromise miRNA function. siRNAs can move through plasmodesmata and act non-cell-autonomously in nearby cells, and RDR6 functions in transitive gene silencing in these neighbor cells [17],[42]. The few neighboring cells adjacent to cell-specific miRNA gene expression might be the source of antisense signals we observe, which could also explain the low abundance signals. As previously suggested [16],[42], coupled miRNA/siRNA mechanisms might function in tissues where the miRNA is not expressed to generate gradients of developmental effectors, e.g. in meristems and primordia, or to allow miRNA activity to be amplified where a limiting amount of miRNA may be present, e.g. in response to stress [67]. Vaucheret *et al.* [68] have shown that minor perturbations of *MIR168* and/or its target *AGO1* expression leads to fine-tuned posttranscriptional adjustment of miR168 and AGO1 levels, thereby maintaining a proper balance of other miRNAs. This suggests that modulating the efficiency of assembling miRNA-programmed RISCs may be important in other contexts or require other determinants. This homeostatic mechanism may help explain our unexpected results on some miRNA target gene antisense transcripts and genotypes (Figures 2–6; also compare Figures 7 and 8). Another possible explanation for the lack of strong effects on antisense and sense miRNA target transcript abundance in *hen1-1* and *sgs3-14* mutants is genetic redundancy, a hallmark of polyploid plant genomes. This hypothesis is congruent with phenotypes of *ago1*, *ago7*, *dcl1*, *hyl1* and *rdr6* mutants that have only modestly altered miRNA and target gene abundances [14],[16],[19],[21],[69], and the existence of parallel genetic pathways for miRNA activity defined by SERRATE, AS1, AS2, and ABH1 [33],[70],[71],[72].

## Materials and Methods

### Plant Growth and RNA Extraction

*Arabidopsis thaliana* seeds were sown to the soil directly, stratified for 72 h at 4°C, and then placed at 21°C under long day condition of 16 h of light. RNA was extracted with TriZol reagent (Invitrogen, Carlsbad CA) or using RNAqueous-Micro isolation kit (Ambion, Austin TX), including the DNAse treatment step, from plants harvested 4 weeks after stratification.

### Design of the Custom Array; Sample Labeling, Hybridization, and Washing; Microarray Scanning, Normalization, and Filtering of Expressed Genes

The protocols for the pilot array experiment are identical to those of Ref. [13]. For 15k arrays with 22 selected miRNA targets, a dye swap loop experiment design was utilized with 12 blocks for 7 genotypes on two chip arrays. The details of the experimental design are in Table S5. Total RNA was isolated from aerial parts of wild type Ler-0 and *hen1-1*, or from inflorescences of wild type Col-0, *dcl1-7*, *hyl1-2*, *sgs3-14* and *rdr6-15*. For the *hen1-1* versus Ler-0 experiment, a dye swap with two versus three biological replicates and four array blocks was performed. After washing, arrays were scanned using a GenePix Autoloader 4200AL with laser excitation at 532 and 635 nm, and saved as 16-bit grayscale TIFF images. Intensity values were extracted using GenePix Pro, and the data for each sample were normalized using standard procedures [73]. Original MIAME-compliant data is stored at the Gene Expression Omnibus (http://www.ncbi.nlm.nih.gov/geo/) with the following locator: GSE15199.

### qRT-PCR Sense and Antisense RNA Expression Analyses

Analyses were done according to standard protocols and manufacturers' instructions except as noted below. Total RNA was treated by RQ1 RNase-free DNase (Promega, Madison WI) and purified with a standard phenol∶chloroform extraction followed by ethanol precipitation. qRT-PCR was performed using M-MLV reverse transcriptase (Promega, Madison WI) with 5 µg total RNA as input for each reaction followed by 32 cycles of PCR and incorporation of α-^32^P-dCTP. ACTIN8 primers were added to the qRT-PCR system as a quantitative internal control for the efficiency of amplification. Products were separated on 12% non-denaturing polyacrylamide gels and results were documented by imaging with a Storm 860 phosphorimager instrument (GE Healthcare, Piscataway NJ). The intensity of signal for the bands on the gels was quantified and normalized by ImageQuant TL software (GE Healthcare). The PCR products that were of the predicted size were the major bands in all experiments, which range from 60 b.p.–220 b.p. To confirm the authenticity for the antisense transcripts of select genes, different controls have been applied in PCR reactions such as control PCR with no primers, with only forward primer or reverse primer, or with no template. The *AP2* PCR products were cloned and sequenced to confirm their identities (data not shown). Primer sequences are shown in Table S10.

### Computational Analysis

Paralogous non-targets for validated miRNA targets were first chosen based on PBLAST scores using the cognate miRNA target gene amino acid sequences for all miRNA families with the highest complementarity and thermodynamic duplex stability scores [74],[75]. The best paralog candidates out of the PBLAST screening were aligned with the corresponding miRNA targets using nucleotide sequence in Vector NTI 9.0 (Invitrogen, Carlsbad CA). The pseudo miRNA binding sites on the paralogs were manually chosen based on the alignment results.

In the statistical analysis of MPSS data, if a gene had no sense expression, a transcripts-per-million value of 1 was given to avoid division by zero in calculating the percentage of antisense expression as a function of total expression. When comparing the signal intensities for validated targets, predicted targets, and paralogous non-targets from previously published whole genome microarray data [11],[13], 95% confidence intervals for the mean values of the signals of 200 n.t. upstream and downstream miRNA binding sites were calculated. The confidence intervals of two different mean values which did not overlap were identified as statistically significantly different. We did not include the confidence intervals for brevity but we assigned different letters to denote statistically different values (See Table S4). smRNA sequences were obtained from published data [20],[28],[37],[38] and were searched against the cDNA sequences (TAIR release 7, ftp://ftp.Arabidopsis.org/home/tair/Sequences/blast_datasets/TAIR7_blastsets/) or miRNA hairpins [76] by the program BLAST. The output sequences were further queried by BLAST against the Arabidopsis genome to find the smRNAs with single loci. All smRNAs matching with known miRNA, miRNA*, or genes previously reported to generate abundant smRNAs including *PPR*, *AGO1*, *ATCHX18*, *ARF2/3/4*, etc. [15],[23] were eliminated from this analysis.

## Supporting Information

Figure S1 Arabidopsis transcriptome profiles (y-axis) for sense (upper panels) and antisense (lower panels) strands of validated miRNA target genes that produce unique smRNAs. The vertical dashed line through the graphs represents the miRNA cleavage site; the asterisks (*) represent cloned unique smRNAs [28],[37]. Arrows show upstream antisense transcripts from 5′ to 3′ direction. The topology of miRNA target gene expression for the 800-n.t. regions flanking the miRNA cleavage site shows a “ping-pong” relationship of strong sense strand expression downstream of, and strong antisense strand expression upstream of, the miRNA cleavage site. (A) *ARF17*/miR160; (B) *AGO2*/miR403; (C) *SCRL6(III)*/miR170; (D) *AP2*/miR172; (E) *GRF3*/miR396; (F) *ARF8*/miR167; (G) *SPL4*/miR157; (H) *TCP4*/miR319; (I) *CHX18*/miR856; (J) *APS1*/miR395; (K) *At5g43740*/miR472; (L) *MET2*/miR773. Line colors indicate RNA samples from T87 callus cultures (blue)[13]; flowers (green); root (magenta); light-grown leaves (brown); and suspension cells (tan) [11]. Exons are denoted as green boxes on the Watson (upper) or Crick (lower) strands (x-axis).
(5.75 MB TIF)

Figure S2 Hybridization signals from custom high resolution microarrays for the sense strand of select miRNA targets transcripts. (A) *AP2*/AT4G36920; (B) *HAP2C*/AT1G72830; (C) *TCP2*/AT4G18390; (D) *AGO2*/AT1G31280; (E) *SPL2*/AT5G43270; (F) *SPL10*/AT1G27370. All data points were from averaged wild type Col-0 samples and plotted as the function of the location of each probe relative to the miRNA cleavage site (zero) on the genome. Blue line indicates the signals from custom tiling microarray using probes of 25-n.t. with the resolution of 3-n.t. Red line displays the signals from custom tiling microarray using probes of 36-n.t. with the resolution of 3-n.t. Green line shows the average signal intensity from five previously published whole genome tiling microarray experiments [11],[13]. Exons or 3′ UTRs for each gene are shown below each plot as green or open boxes, respectively. Introns are indicated by straight lines and the intergenic region is denoted by dashed line.
(0.88 MB TIF)

Figure S3 Increased transcription signals from custom tiling microarray for *hen1-1* mutant versus wild type Ler-0 (x axis) were correlated with previously published Affymetrix ATH1 microarray data (y axis) [19]. Lines represent best-fit linear regression; R^2^ values represent Pearson correlation coefficients.
(0.13 MB TIF)

Figure S4 Hybridization signals for the antisense strand of select miRNA targets transcripts. (A) *APS1*/AT3G22890; (B) *MYB12*/AT2G47460; (C) *SCR6(IV)*/AT4G00150; (D) *TOE2*/AT5G60120; (E) *AP2*/AT4G36920; (F) *GRF8*/AT4G24150. All data points are plotted as the function of the location of each probe relative to the miRNA cleavage site (zero) on the genome. Blue line indicates the average signals for wild type Ler-0 from two custom tiling microarrays using probes of 25- and 36-n.t. with the resolution of 3-n.t. Red line displays the signals for wild type Col-0 from the same two custom tiling microarrays as those for Ler-0.
(0.57 MB TIF)

Figure S5 Normalized antisense transcript delta signals for a validated miRNA target, *APS1*/AT3G22890. Each data point is the average signal of at least 3 technical samples and is represented by the difference between the signals from different mutants versus their corresponding wild type control, divided by that from the control [normalized “delta” Δ signal = (mutant signal-wild type signal)/wild type signal]. Ler-0 is the control for *hen1-1* mutant, while Col-0 is the control for *dcl1-7*, *hyl1-2*, *rdr6-15* and *sgs3-14*. The normalized delta signal is plotted as a function of probe position relative to the miRNA cleavage site (coordinate zero on x-axis). Black arrow indicates the changed signals identified by probe sets with at least 3 contiguous probes showing at least 20% differences (up or down, not both) for the signal changes in the mutant versus that of wild type. The precise same region with changed signals, if any, is indicated by black arrows for other smRNA mutants in Figures S6, S7, S8, S9, S10, S11, S12, S13, S14, S15, S16.
(0.78 MB TIF)

Figure S6 Normalized antisense transcript delta signals for a validated miRNA target, *MYB12*/AT2G47460. See Fig. S5 for details of legend. The open arrow pinpoints the decreased antisense signal adjacent to the increased antisense signals in *sgs3-14* mutants.
(0.80 MB TIF)

Figure S7 Normalized antisense transcript delta signals for a validated miRNA target, *AP2*/AT4G36920. See Fig. S5 for details of legend.
(0.81 MB TIF)

Figure S8 Normalized antisense transcript delta signals for a validated miRNA target, *GRF8*/AT4G24150. See Fig. S5 for details of legend.
(0.88 MB TIF)

Figure S9 Normalized antisense transcript delta signals for a validated miRNA target, *SCL6(IV)*/AT4G00150. See Fig. S5 for details of legend.
(0.78 MB TIF)

Figure S10 Normalized antisense transcript delta signals for a validated miRNA target, *TOE2*/AT5G60120. See Fig. S5 for details of legend.
(0.77 MB TIF)

Figure S11 Normalized antisense transcript delta signals for a validated miRNA target, *DCL1*/AT1G01040. See Fig. S5 for details of legend.
(0.90 MB TIF)

Figure S12 Normalized antisense transcript delta signals for a validated miRNA target, *SPL10*/AT1G27370. See Fig. S5 for details of legend.
(0.77 MB TIF)

Figure S13 Normalized antisense transcript delta signals for a validated miRNA target, *MET2*/AT4G14140. See Fig. S5 for details of legend.
(0.81 MB TIF)

Figure S14 Normalized antisense transcript delta signals for a validated miRNA target, *TCP4*/AT3G15030. See Fig. S5 for details of legend. The open arrow pinpoints the significantly decreased antisense signal adjacent to the significantly increased antisense signals in *sgs3-14* mutants.
(0.68 MB TIF)

Figure S15 Normalized antisense transcript delta signals for a validated miRNA target, *UBC24*/AT2G33770. See Fig. S5 for details of legend.
(0.88 MB TIF)

Figure S16 Normalized antisense transcript delta signals for a validated miRNA target, *CC-NBS-LRR*/AT5G43740. See Fig. S5 for details of legend.
(0.87 MB TIF)

Figure S17 Fraction of small RNAs mapping to the mature miRNA or miRNA* sites on miRNA hairpins. smRNA sequences were obtained from published deep sequencing data [20],[28],[37],[38]. Unique smRNAs with perfect matches to miRNA hairpins (http://microrna.sanger.ac.uk) were found by the BLAST program. Open bar indicates the percentage of unique smRNAs with at least 16 n.t. overlap to mature miRNAs on the sense strand or to the opposite location on the antisense stand of miRNA hairpins, while black bar displays the percentage of the unique smRNAs with at least 16 n.t. overlap to the miRNA* sites on the sense strand or to the opposite location on the antisense strand of miRNA hairpins.
(0.18 MB TIF)

Figure S18 Antisense phased smRNAs mapping to miRNA hairpin sequences. (A) miR783 hairpin sequence. (B) miR854b hairpin sequence. smRNA sequences were obtained as described in Fig. S17 legend. The mature miRNA site on the miRNA hairpin is underlined by red line, while the miRNA* site is indicated by blue line. Cloned smRNAs are labeled by their database names and lengths from individual sources with solid brackets above the hairpin sequence. Predicted smRNAs are indicated by dashed brackets. #: small RNAs from [28]; ‡: small RNAs from [37]; †: small RNAs from [38].
(0.34 MB TIF)

Table S1 General information on miRNA targets and paralog genes.
(0.11 MB XLS)

Table S2 MPSS mRNA signatures associated with miRNA targets and paralogous non-target genes.
(0.57 MB XLS)

Table S3 Previously published whole genome tiling microarray data for miRNA targets, paralogous non-targets and *MIRNA* genes.
(15.17 MB XLS)

Table S4 Statistical analysis of previously published whole genome tiling microarray data for miRNA targets and paralogous non-target genes.
(0.02 MB XLS)

Table S5 Experimental design for custom tiling microarrays.
(0.13 MB XLS)

Table S6 Raw signals from the antisense strand of 22 validated miRNA targets on the custom high resolution tiling microarrays.
(1.67 MB XLS)

Table S7 Normalized delta sense and antisense signals for 22 validated miRNA targets on the custom high resolution tiling microarrays.
(3.45 MB XLS)

Table S8 smRNAs with perfect match to miRNA targets, paralogous non-targets and MIRNA hairpins.
(1.30 MB XLS)

Table S9 Location of MPSS smRNA signatures with perfect match to miRNA targets and paralogous non-target genes.
(0.30 MB XLS)

Table S10 Primers used for strand-specific semi-quantitative qRT-PCR in this study.
(0.02 MB XLS)

Text S1 Supplemental materials and methods.
(0.04 MB DOC)
