# Supplementary figures and images for: Evidence for Antisense Transcription Associated with MicroRNA Target mRNAs in Arabidopsis
Source: PLoS Genet. 2009 Apr 17;5(4):e1000457. doi: 10.1371/journal.pgen.1000457 (PMC2664332; doi:10.1371/journal.pgen.1000457)

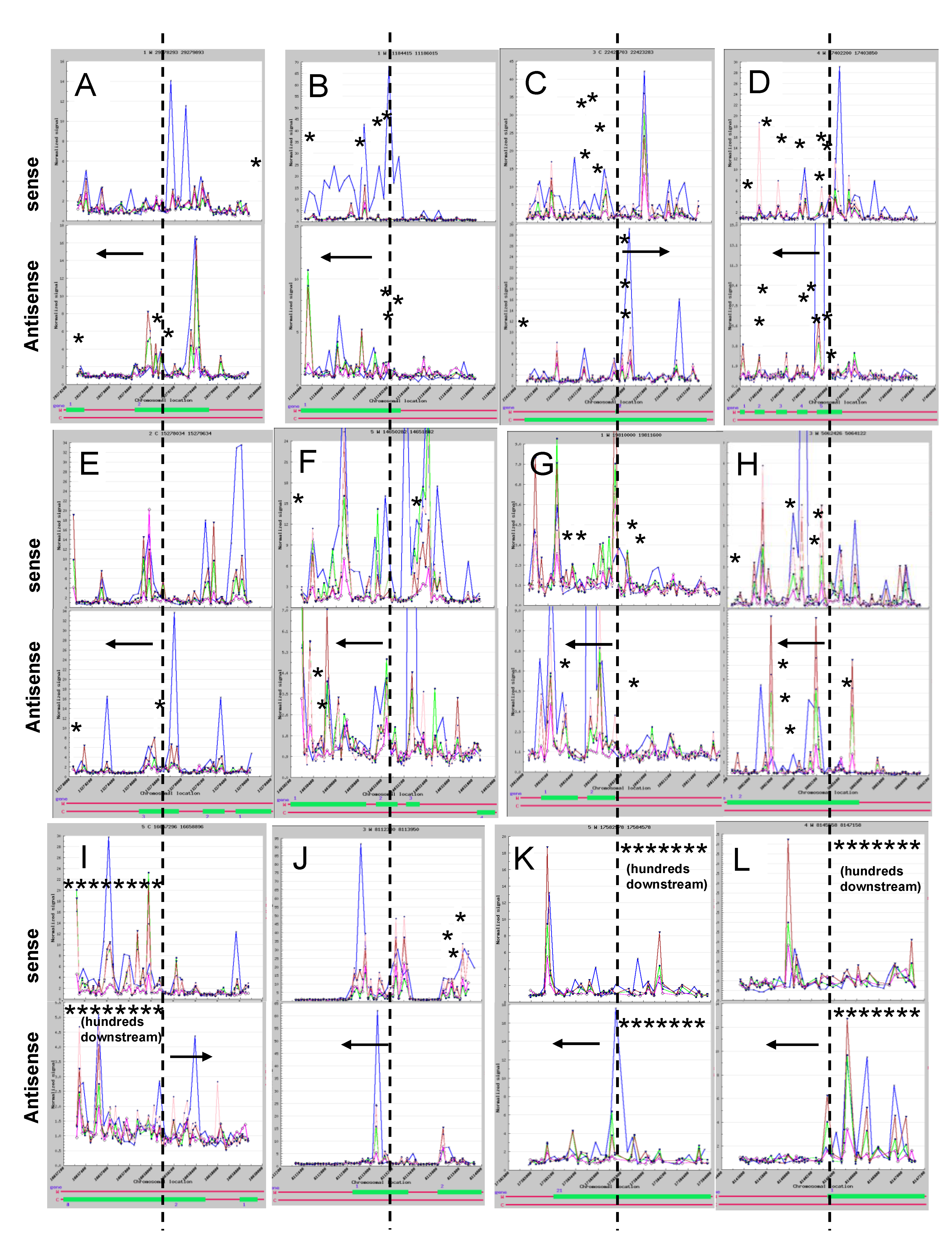

Supplement: Figure S1 — Arabidopsis transcriptome profiles (y-axis) for sense (upper panels) and antisense (lower panels) strands of validated miRNA target genes that produce unique smRNAs. The vertical dashed line through the graphs represents the miRNA cleavage site; the asterisks (*) represent cloned unique smRNAs [28],[37]. Arrows show upstream antisense transcripts from 5′ to 3′ direction. The topology of miRNA target gene expression for the 800-n.t. regions flanking the miRNA cleavage site shows a “ping-pong” relationship of strong sense strand expression downstream of, and strong antisense strand expression upstream of, the miRNA cleavage site. (A) ARF17/miR160; (B) AGO2/miR403; (C) SCRL6(III)/miR170; (D) AP2/miR172; (E) GRF3/miR396; (F) ARF8/miR167; (G) SPL4/miR157; (H) TCP4/miR319; (I) CHX18/miR856; (J) APS1/miR395; (K) At5g43740/miR472; (L) MET2/miR773. Line colors indicate RNA samples from T87 callus cultures (blue)[13]; flowers (green); root (magenta); light-grown leaves (brown); and suspension cells (tan) [11]. Exons are denoted as green boxes on the Watson (upper) or Crick (lower) strands (x-axis). (5.75 MB TIF) [file pgen.1000457.s001.tif]

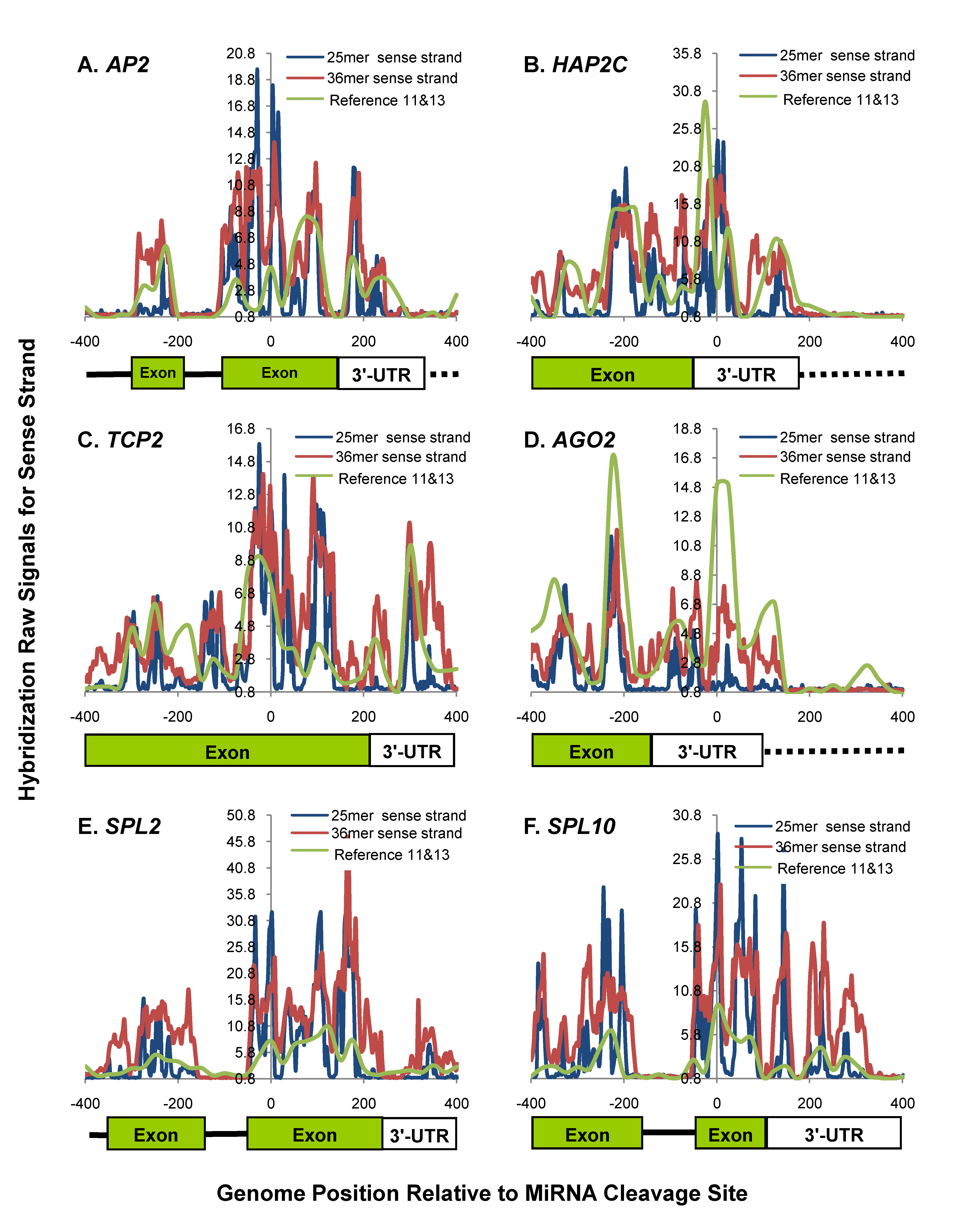

Supplement: Figure S2 — Hybridization signals from custom high resolution microarrays for the sense strand of select miRNA targets transcripts. (A) AP2/AT4G36920; (B) HAP2C/AT1G72830; (C) TCP2/AT4G18390; (D) AGO2/AT1G31280; (E) SPL2/AT5G43270; (F) SPL10/AT1G27370. All data points were from averaged wild type Col-0 samples and plotted as the function of the location of each probe relative to the miRNA cleavage site (zero) on the genome. Blue line indicates the signals from custom tiling microarray using probes of 25-n.t. with the resolution of 3-n.t. Red line displays the signals from custom tiling microarray using probes of 36-n.t. with the resolution of 3-n.t. Green line shows the average signal intensity from five previously published whole genome tiling microarray experiments [11],[13]. Exons or 3′ UTRs for each gene are shown below each plot as green or open boxes, respectively. Introns are indicated by straight lines and the intergenic region is denoted by dashed line. (0.88 MB TIF) [file pgen.1000457.s002.tif]

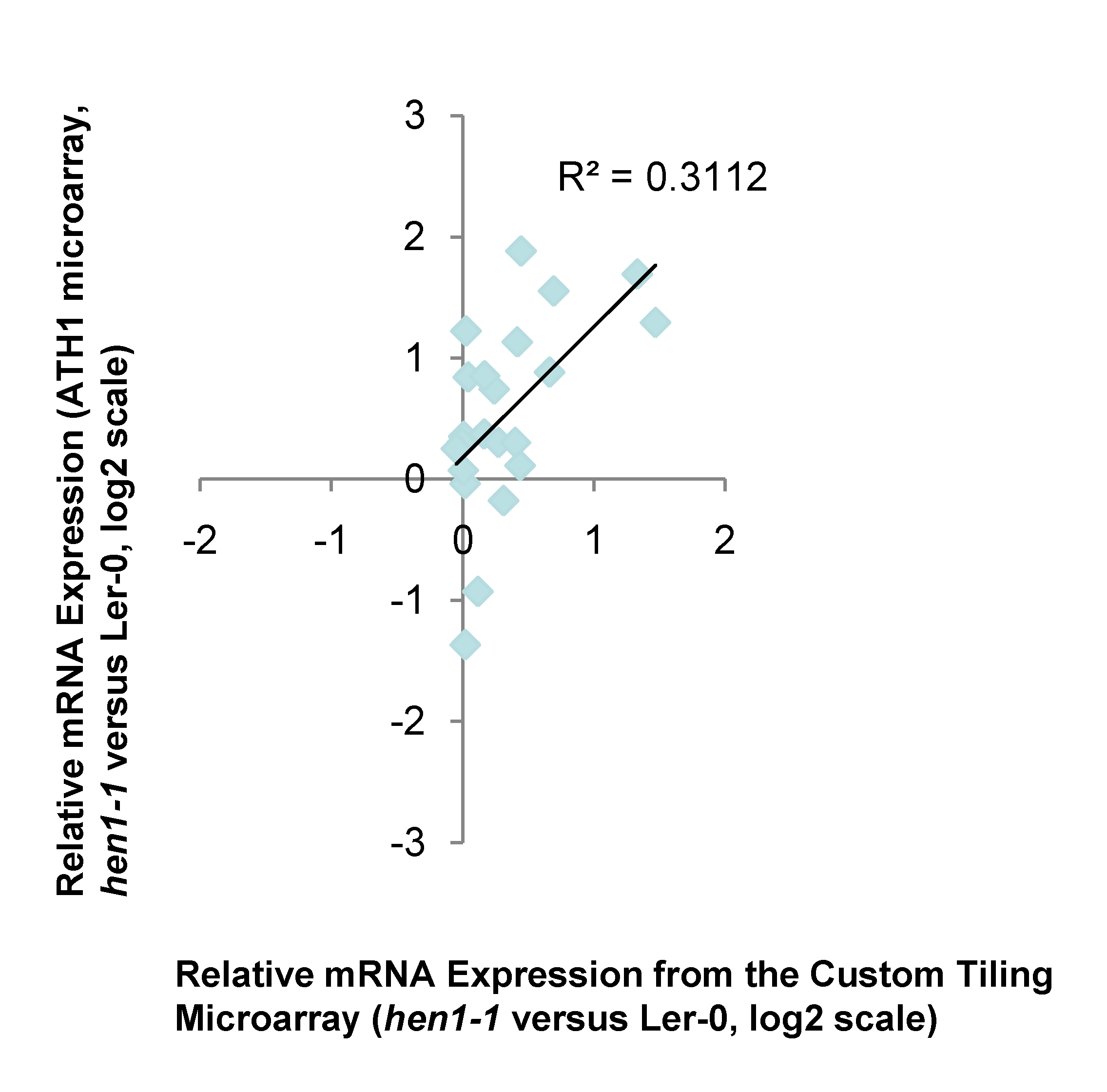

Supplement: Figure S3 — Increased transcription signals from custom tiling microarray for hen1-1 mutant versus wild type Ler-0 (x axis) were correlated with previously published Affymetrix ATH1 microarray data (y axis) [19]. Lines represent best-fit linear regression; R2 values represent Pearson correlation coefficients. (0.13 MB TIF) [file pgen.1000457.s003.tif]

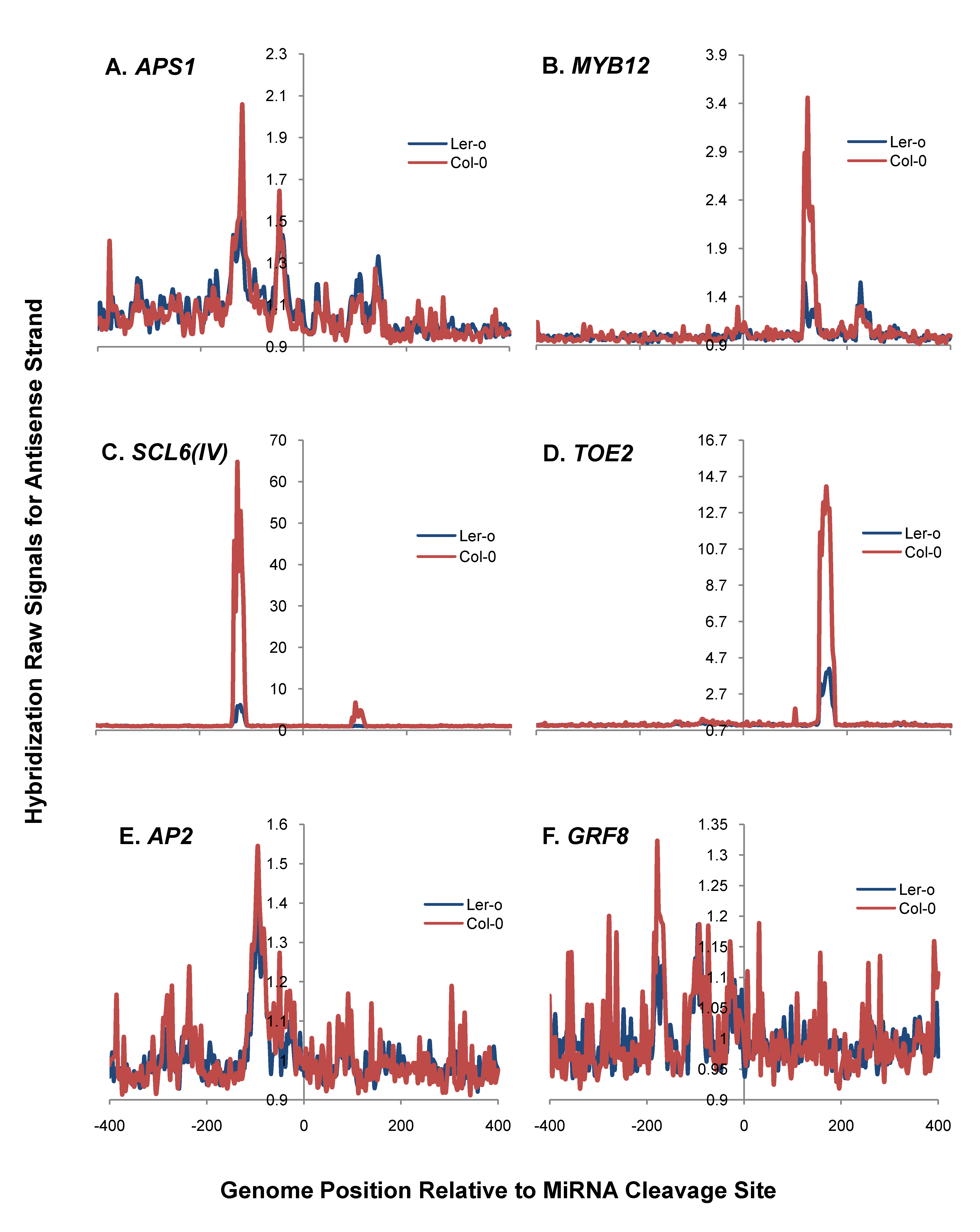

Supplement: Figure S4 — Hybridization signals for the antisense strand of select miRNA targets transcripts. (A) APS1/AT3G22890; (B) MYB12/AT2G47460; (C) SCR6(IV)/AT4G00150; (D) TOE2/AT5G60120; (E) AP2/AT4G36920; (F) GRF8/AT4G24150. All data points are plotted as the function of the location of each probe relative to the miRNA cleavage site (zero) on the genome. Blue line indicates the average signals for wild type Ler-0 from two custom tiling microarrays using probes of 25- and 36-n.t. with the resolution of 3-n.t. Red line displays the signals for wild type Col-0 from the same two custom tiling microarrays as those for Ler-0. (0.57 MB TIF) [file pgen.1000457.s004.tif]

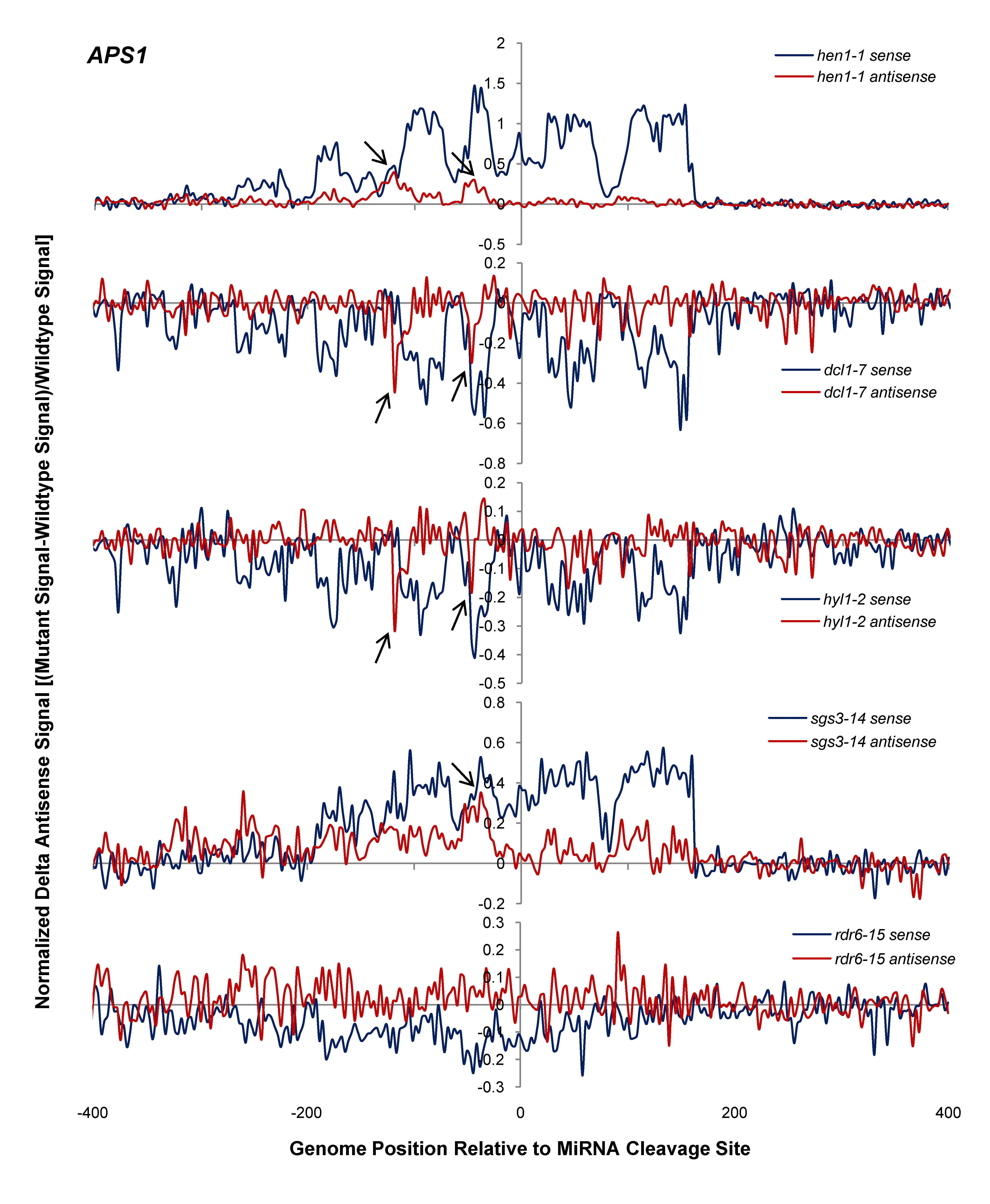

Supplement: Figure S5 — Normalized antisense transcript delta signals for a validated miRNA target, APS1/AT3G22890. Each data point is the average signal of at least 3 technical samples and is represented by the difference between the signals from different mutants versus their corresponding wild type control, divided by that from the control [normalized “delta” Δ signal = (mutant signal-wild type signal)/wild type signal]. Ler-0 is the control for hen1-1 mutant, while Col-0 is the control for dcl1-7, hyl1-2, rdr6-15 and sgs3-14. The normalized delta signal is plotted as a function of probe position relative to the miRNA cleavage site (coordinate zero on x-axis). Black arrow indicates the changed signals identified by probe sets with at least 3 contiguous probes showing at least 20% differences (up or down, not both) for the signal changes in the mutant versus that of wild type. The precise same region with changed signals, if any, is indicated by black arrows for other smRNA mutants in Figures S6, S7, S8, S9, S10, S11, S12, S13, S14, S15, S16. (0.78 MB TIF) [file pgen.1000457.s005.tif]

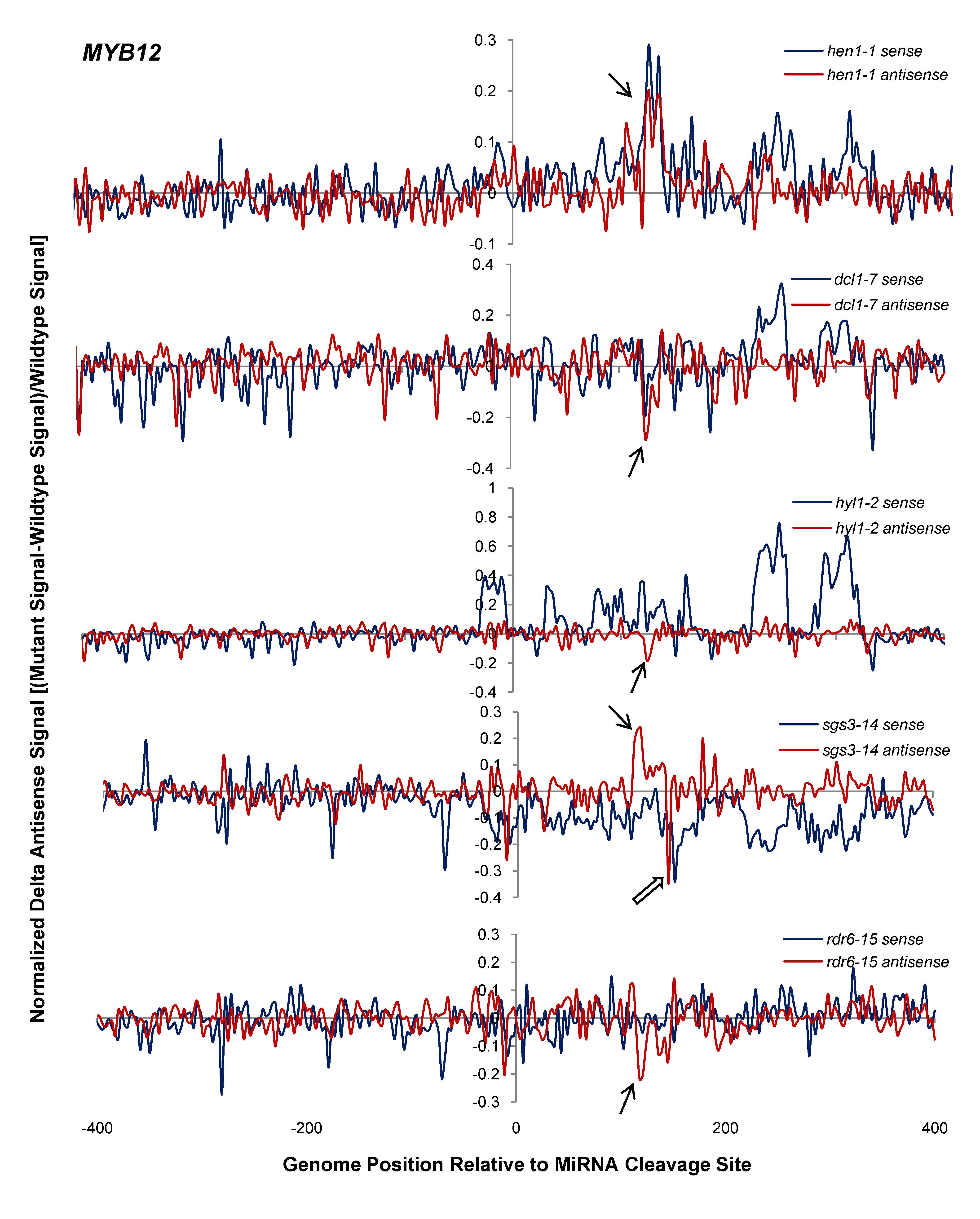

Supplement: Figure S6 — Normalized antisense transcript delta signals for a validated miRNA target, MYB12/AT2G47460. See Fig. S5 for details of legend. The open arrow pinpoints the decreased antisense signal adjacent to the increased antisense signals in sgs3-14 mutants. (0.80 MB TIF) [file pgen.1000457.s006.tif]

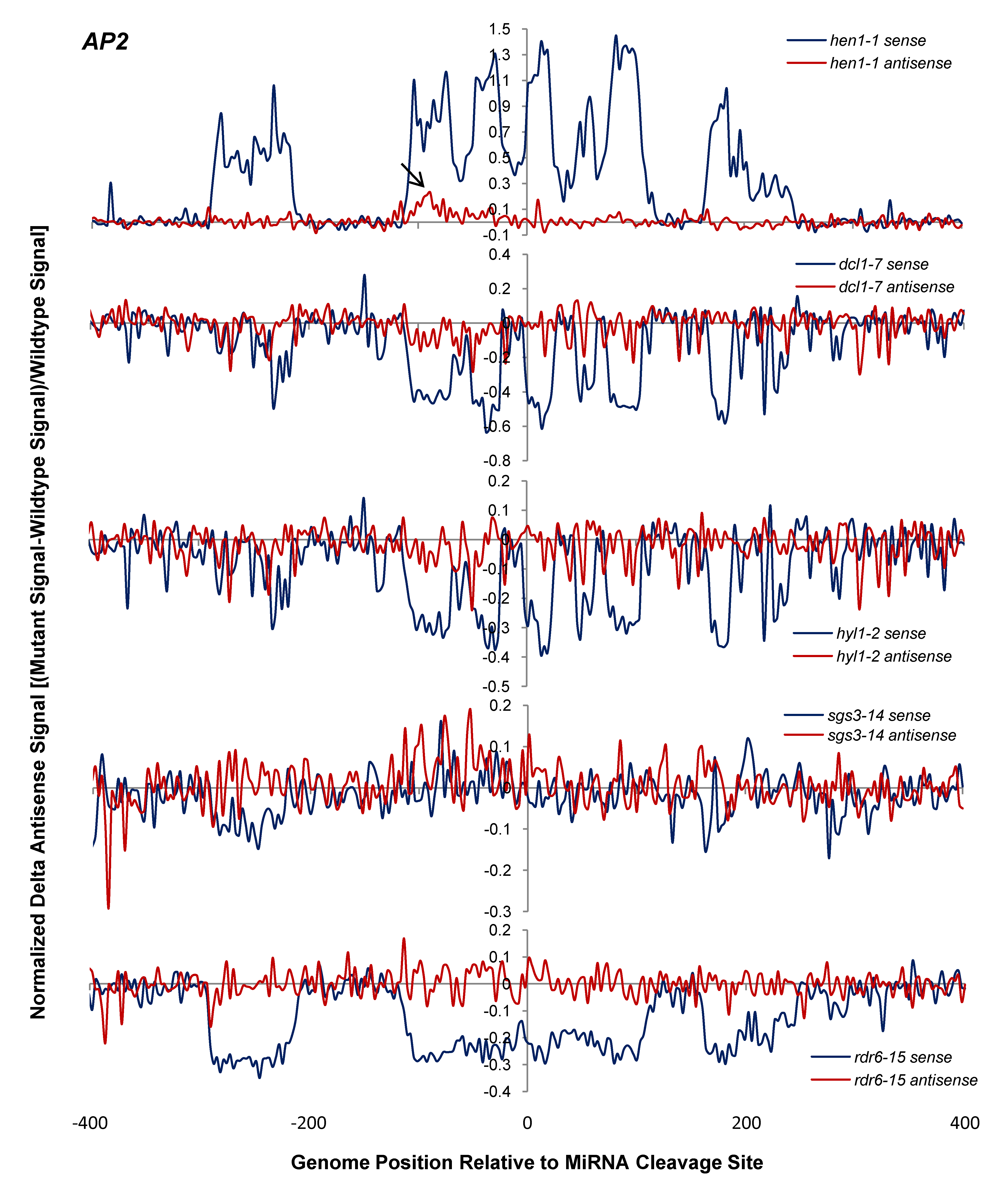

Supplement: Figure S7 — Normalized antisense transcript delta signals for a validated miRNA target, AP2/AT4G36920. See Fig. S5 for details of legend. (0.81 MB TIF) [file pgen.1000457.s007.tif]

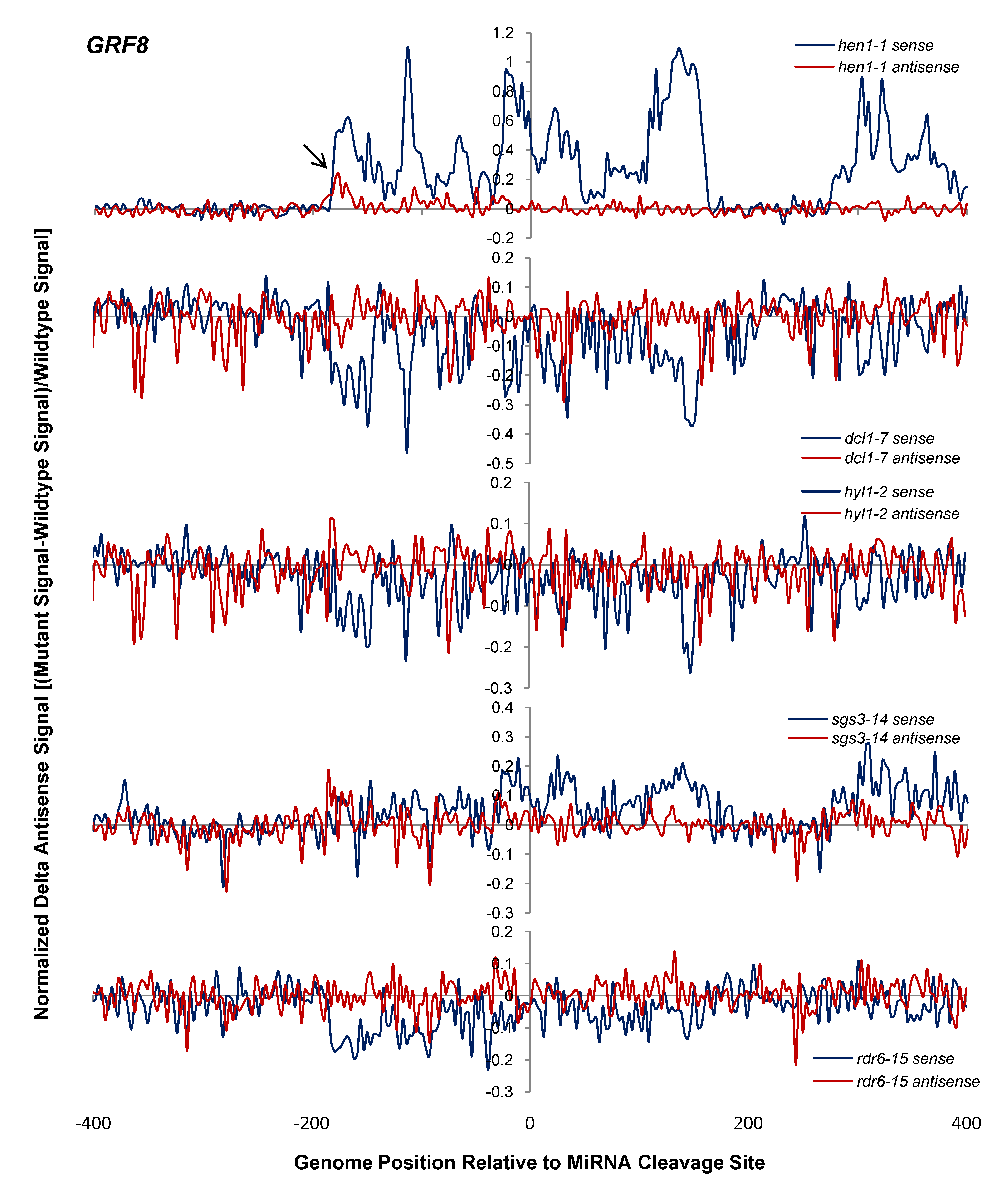

Supplement: Figure S8 — Normalized antisense transcript delta signals for a validated miRNA target, GRF8/AT4G24150. See Fig. S5 for details of legend. (0.88 MB TIF) [file pgen.1000457.s008.tif]

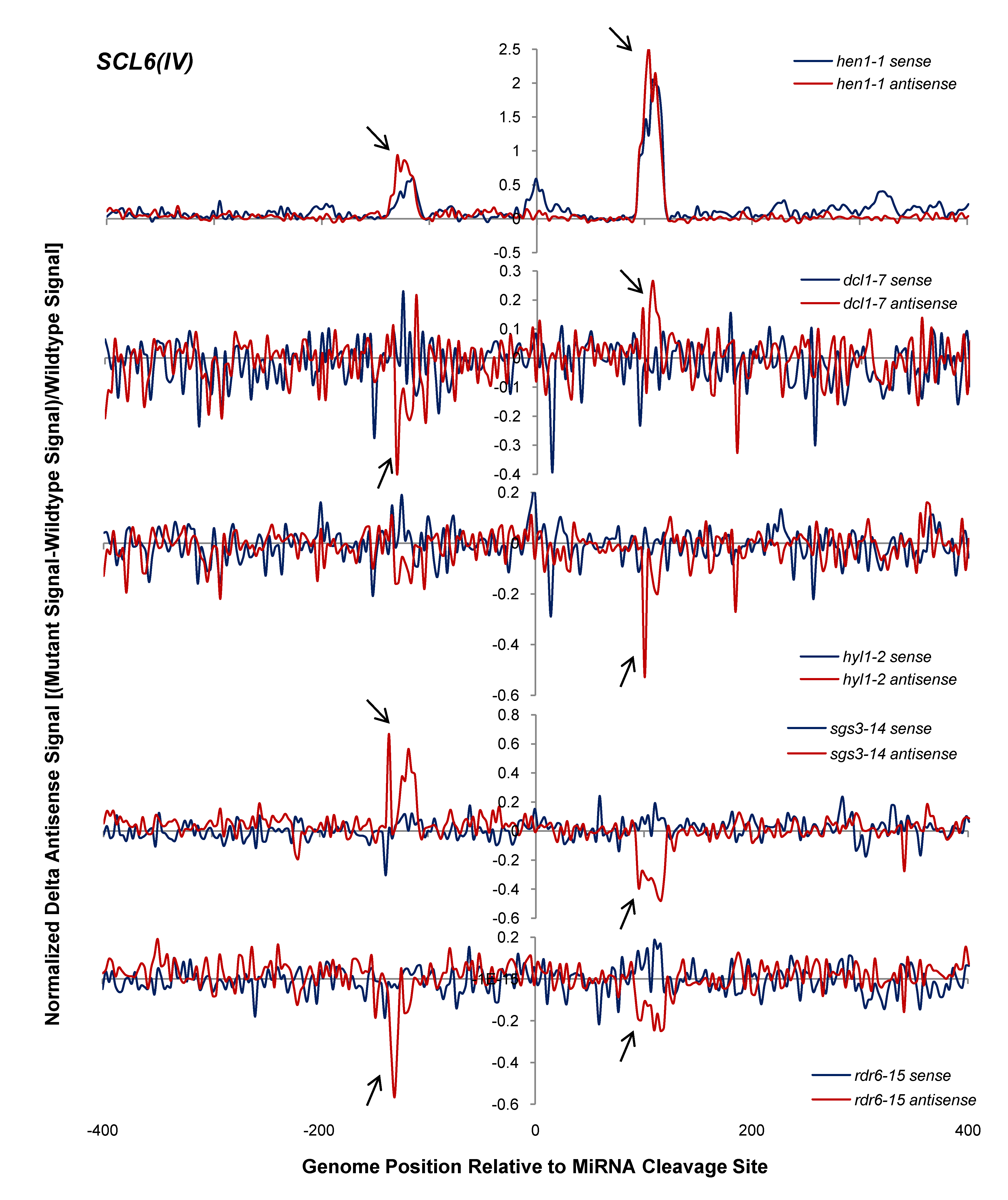

Supplement: Figure S9 — Normalized antisense transcript delta signals for a validated miRNA target, SCL6(IV)/AT4G00150. See Fig. S5 for details of legend. (0.78 MB TIF) [file pgen.1000457.s009.tif]

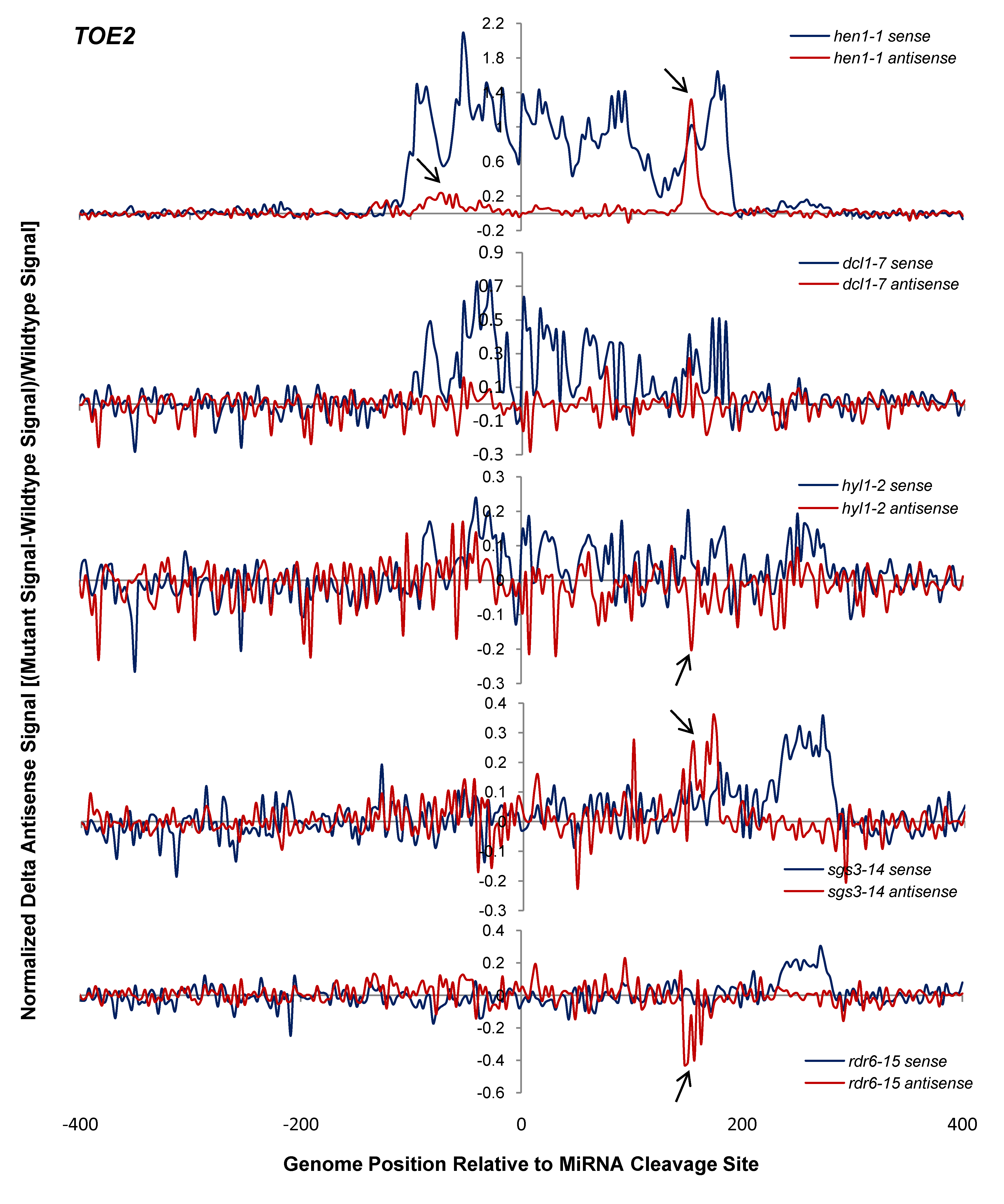

Supplement: Figure S10 — Normalized antisense transcript delta signals for a validated miRNA target, TOE2/AT5G60120. See Fig. S5 for details of legend. (0.77 MB TIF) [file pgen.1000457.s010.tif]

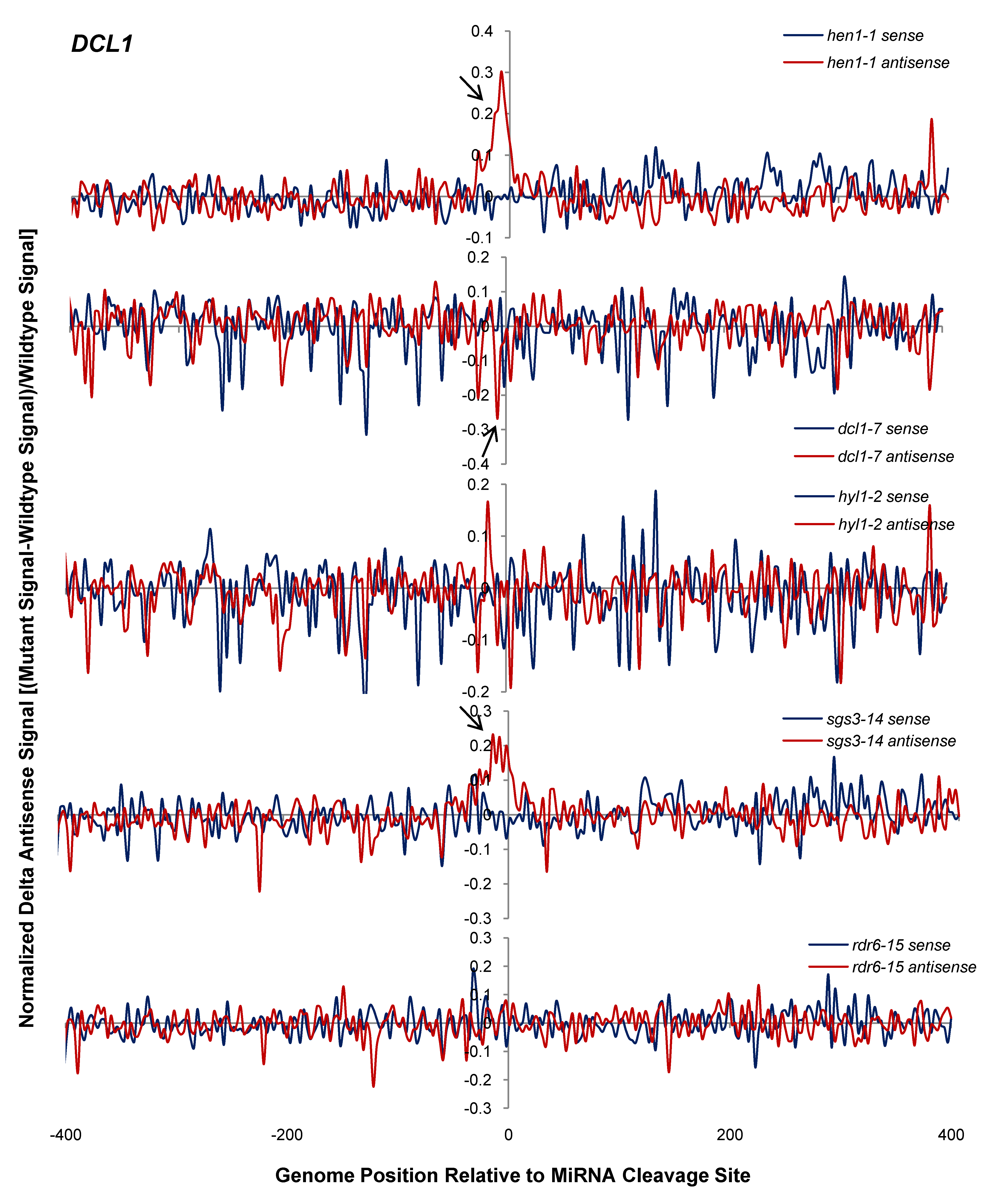

Supplement: Figure S11 — Normalized antisense transcript delta signals for a validated miRNA target, DCL1/AT1G01040. See Fig. S5 for details of legend. (0.90 MB TIF) [file pgen.1000457.s011.tif]

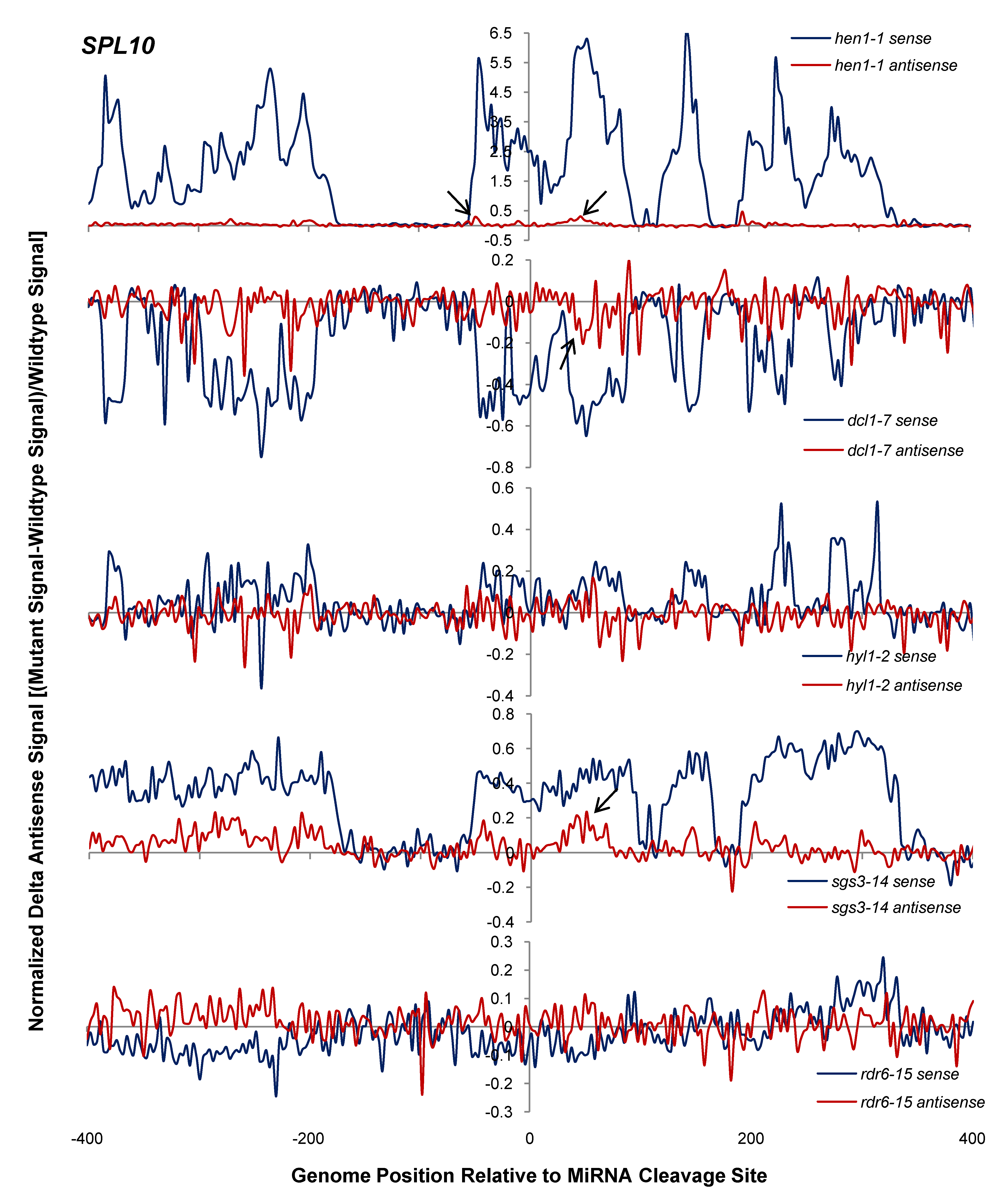

Supplement: Figure S12 — Normalized antisense transcript delta signals for a validated miRNA target, SPL10/AT1G27370. See Fig. S5 for details of legend. (0.77 MB TIF) [file pgen.1000457.s012.tif]

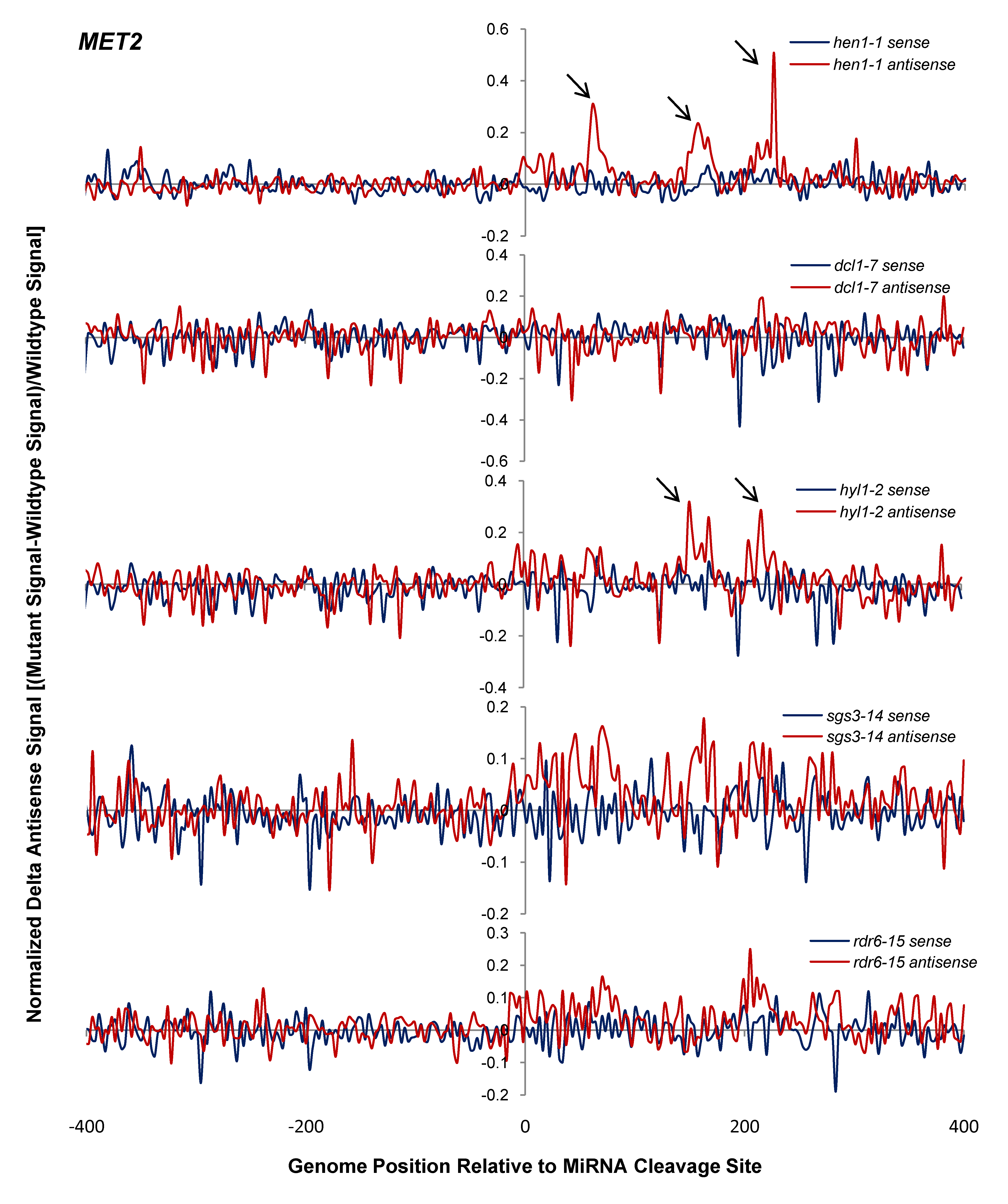

Supplement: Figure S13 — Normalized antisense transcript delta signals for a validated miRNA target, MET2/AT4G14140. See Fig. S5 for details of legend. (0.81 MB TIF) [file pgen.1000457.s013.tif]

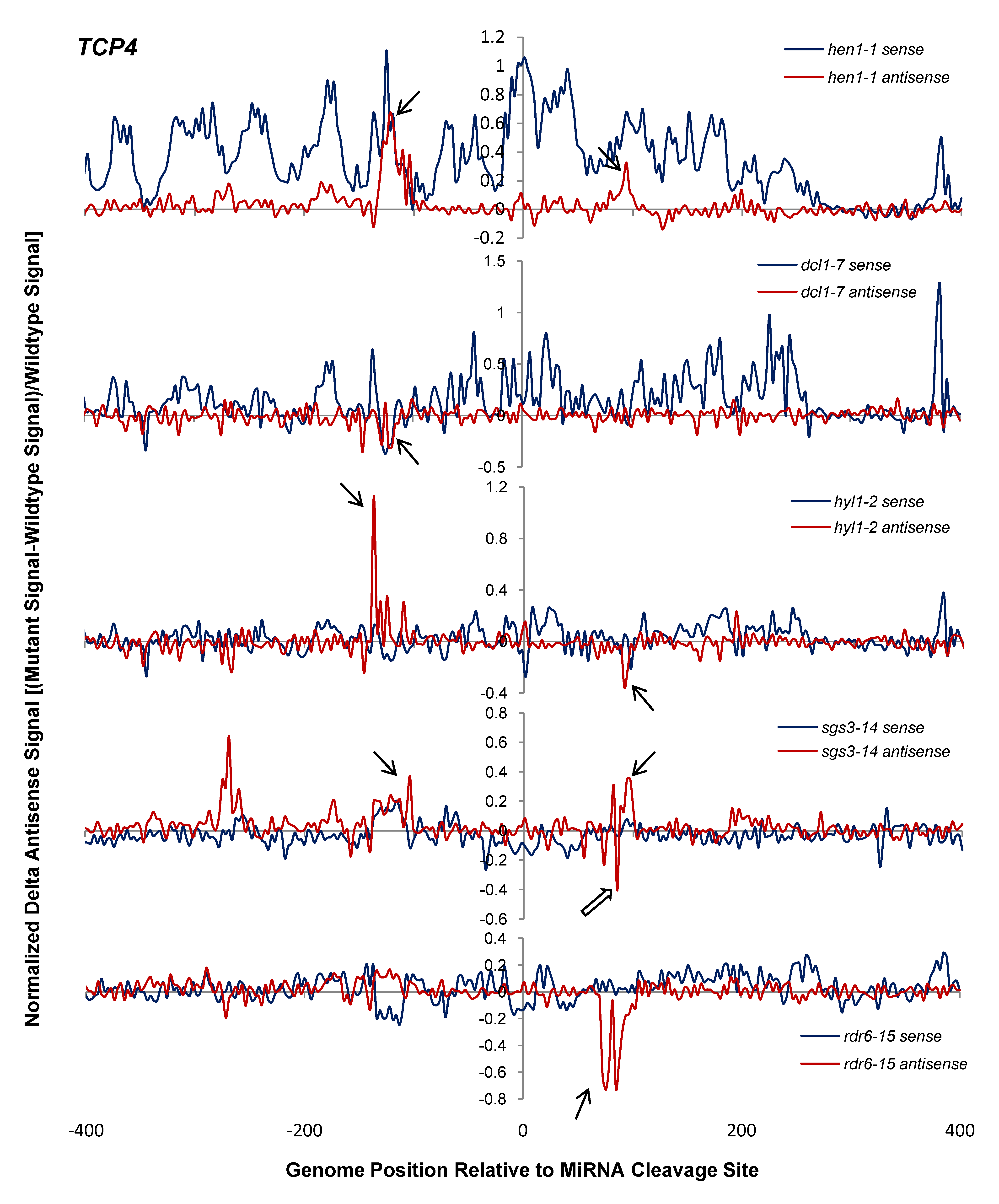

Supplement: Figure S14 — Normalized antisense transcript delta signals for a validated miRNA target, TCP4/AT3G15030. See Fig. S5 for details of legend. The open arrow pinpoints the significantly decreased antisense signal adjacent to the significantly increased antisense signals in sgs3-14 mutants. (0.68 MB TIF) [file pgen.1000457.s014.tif]

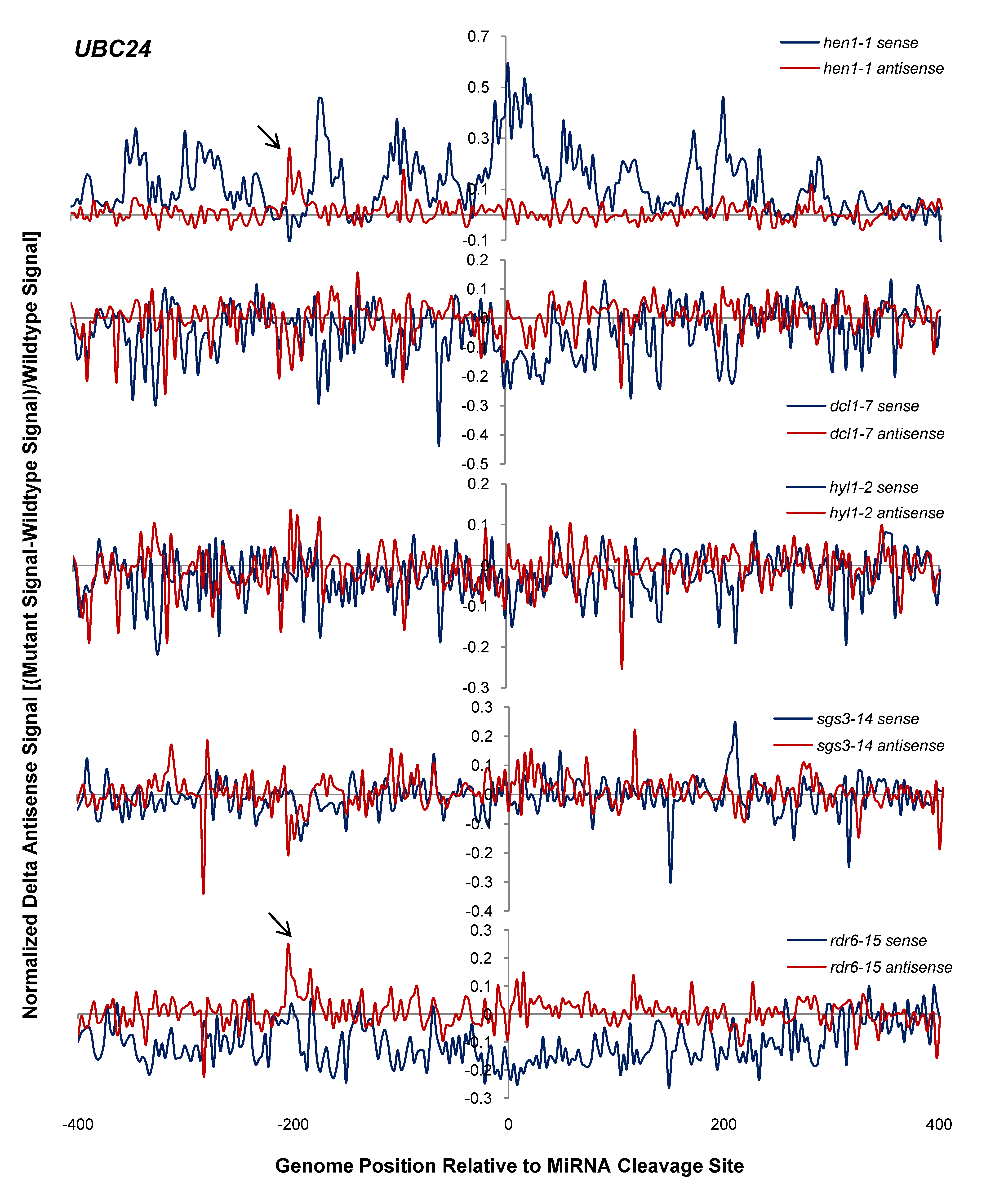

Supplement: Figure S15 — Normalized antisense transcript delta signals for a validated miRNA target, UBC24/AT2G33770. See Fig. S5 for details of legend. (0.88 MB TIF) [file pgen.1000457.s015.tif]

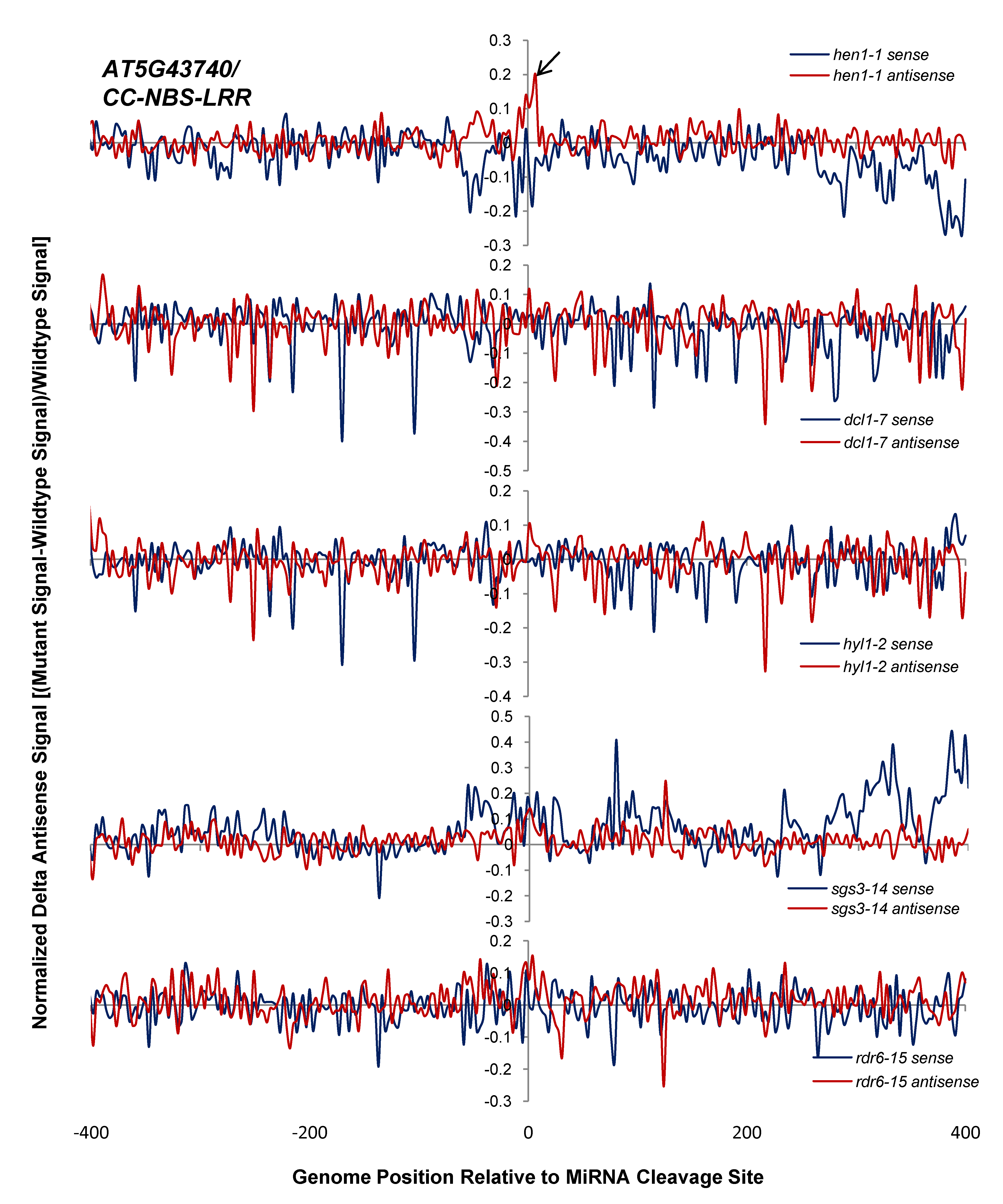

Supplement: Figure S16 — Normalized antisense transcript delta signals for a validated miRNA target, CC-NBS-LRR/AT5G43740. See Fig. S5 for details of legend. (0.87 MB TIF) [file pgen.1000457.s016.tif]

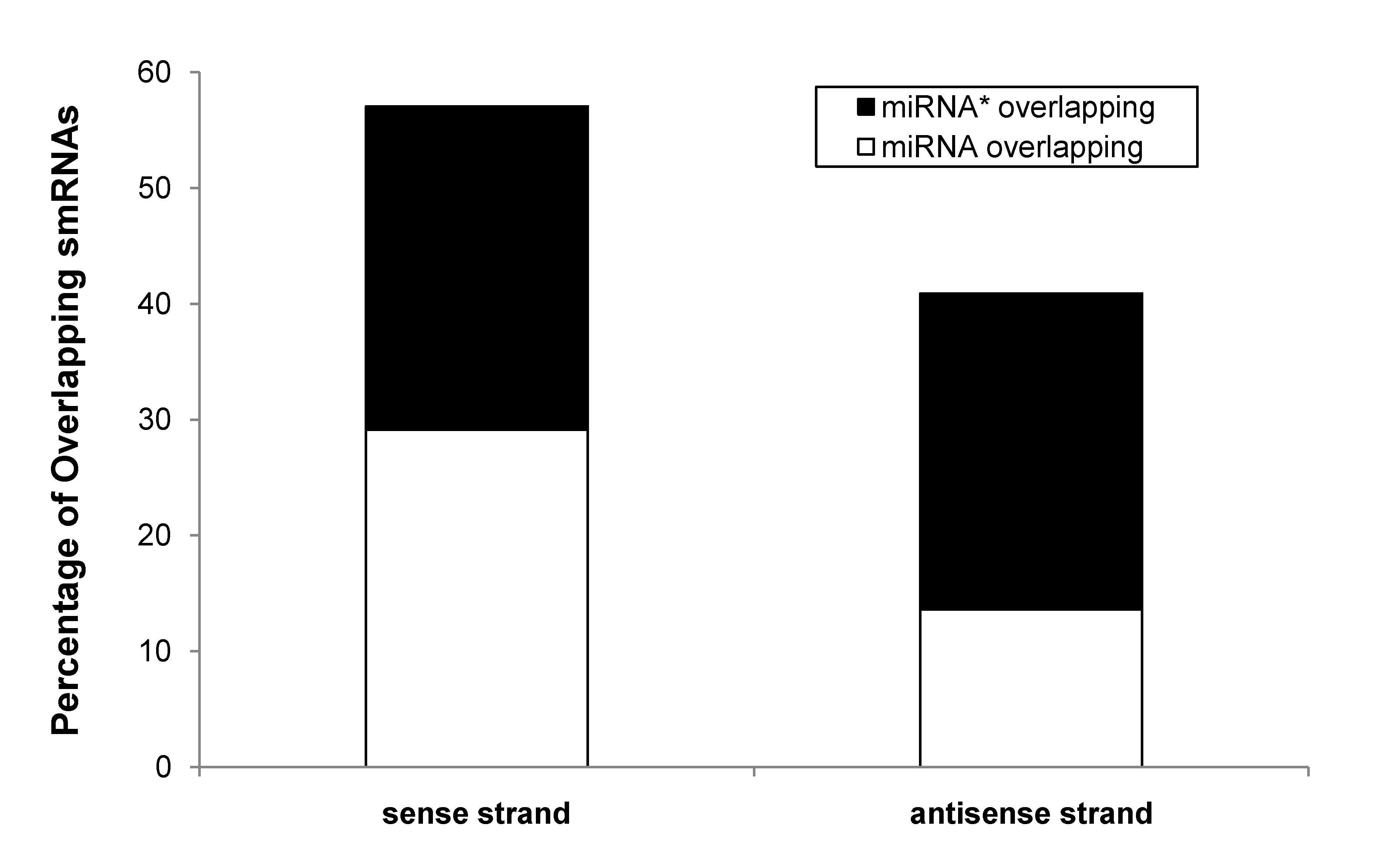

Supplement: Figure S17 — Fraction of small RNAs mapping to the mature miRNA or miRNA* sites on miRNA hairpins. smRNA sequences were obtained from published deep sequencing data [20],[28],[37],[38]. Unique smRNAs with perfect matches to miRNA hairpins (http://microrna.sanger.ac.uk) were found by the BLAST program. Open bar indicates the percentage of unique smRNAs with at least 16 n.t. overlap to mature miRNAs on the sense strand or to the opposite location on the antisense stand of miRNA hairpins, while black bar displays the percentage of the unique smRNAs with at least 16 n.t. overlap to the miRNA* sites on the sense strand or to the opposite location on the antisense strand of miRNA hairpins. (0.18 MB TIF) [file pgen.1000457.s017.tif]

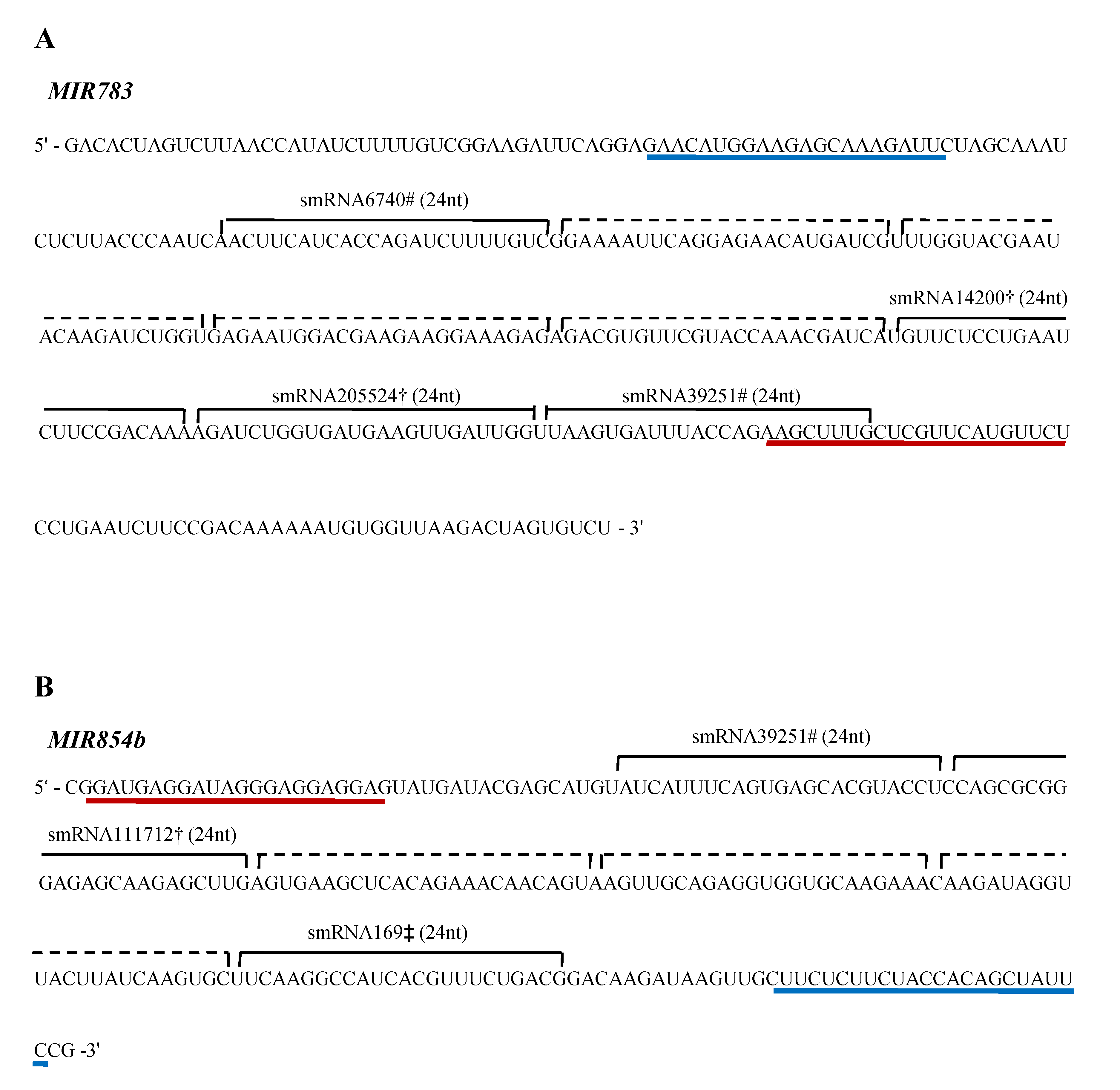

Supplement: Figure S18 — Antisense phased smRNAs mapping to miRNA hairpin sequences. (A) miR783 hairpin sequence. (B) miR854b hairpin sequence. smRNA sequences were obtained as described in Fig. S17 legend. The mature miRNA site on the miRNA hairpin is underlined by red line, while the miRNA* site is indicated by blue line. Cloned smRNAs are labeled by their database names and lengths from individual sources with solid brackets above the hairpin sequence. Predicted smRNAs are indicated by dashed brackets. #: small RNAs from [28]; ‡: small RNAs from [37]; †: small RNAs from [38]. (0.34 MB TIF) [file pgen.1000457.s018.tif]
