# Supplementary material for: Evidence for Antisense Transcription Associated with MicroRNA Target mRNAs in Arabidopsis
Source: PLoS Genet. 2009 Apr 17;5(4):e1000457. doi: 10.1371/journal.pgen.1000457 (PMC2664332; doi:10.1371/journal.pgen.1000457)
Supplement: Text S1 — Supplemental materials and methods. (0.04 MB DOC) [file pgen.1000457.s029.doc]

**Text S1**

**Statistical Analysis of Previously Published Whole Genome Tiling Microarray Data**

The signal from every 25-n.t. region in every one of the five samples [11,13] was divided by the aggregate signal for the 800-n.t. region spanning (5’ upstream and 3’ downstream) the validated, predicted, paralog remnant, or MIRNA* miRNA target sites. The normalized signal was then plotted as a function of the relative position for the miRNA target site. The results are shown in Figure 1. Each data point on the plot was normalized internally (per gene) as a percentage of total hybridization signal integrated over the region of interest for both sense and antisense strands.

**Pilot Tiling Microarray Experiments to Validate the Authenticity of Antisense Transcripts Associated with miRNA Targets**

Two pilot validation experiments for existence of antisense transcripts of miRNA target genes were performed according to previous protocols [13]. Independently amplified copy RNA samples from tissue-cultured Arabidopsis cells and wild type Columbia whole plant total RNAs were hybridized to custom high-resolution (3 n.t. or 20 n.t. spacing) tiling microarrays synthesized using maskless array technology [79]. The probes spanned the 30 n.t. regions immediately upstream and downstream of 123 miRNA binding sites for the 3-n.t. resolution chip. In the second experiment (with 20 n.t. resolution), 400-n.t. upstream and downstream of 120 known and predicted miRNA binding sites for their targets, plus ten experimentally verified non-miRNA targets, were probed [19,27]. Using different labeling schemes, we confirmed that the measured antisense activities are technology-independent (data not shown). Results showed significantly more antisense hybridization signals on the complementary region 5’ upstream than the complementary region 3’ downstream of a miRNA binding site for the validated and predicted targets (data not shown). For the ten validated non-miRNA targets placed on the custom microarray as biological controls, no such correlation was observed.

**Transcript Profiling with a Custom High Resolution Microarray for Select miRNA Target Genes**

To test the hypotheses that miRNA or RNA silencing pathways can affect antisense transcription, we designed two custom high resolution (3 n.t.) tiling microarrays for select miRNA target genes. smRNA pathway mutants *hen1-1, hyl1-2, dcl1-7*, *rdr6-15* and *sgs3-14*, were chosen for analysis. The microarray design incorporated both 25mer and 36mer probe lengths (60mers with a spacer, 8 x 15k features; Agilent Technologies, Santa Clara, CA) spanning the 400-n.t. 5’ upstream and 3’ downstream of miRNA binding sites for 22 validated miRNA targets. The normalized hybridization signals detected from independently isolated and amplified total RNA samples were compared between 25mer and 36mer microarrays and to previously published data to address the quality of the microarrays. The Pearson correlation for all ~44,000 data points technically replicated between 25mer and 36mer chips was 0.84, which established the reproducibility of our experiment and justified combining the biological and technical replicate data from the two microarrays (see [www.ncbi.nlm.nih.gov/geo/](http://www.ncbi.nlm.nih.gov/geo/), locator # GSE15199). Figure S2 shows reproducible hybridization signals for the sense strand of six genes obtained from 25mer and 36mer microarrays and compared directly with the average signal intensity of five experiments previously published [11,13]. It was evident that the signal is a smoother function for the 36mers than for 25mers, as predicted by theory. The signal also appeared stronger for the 36mer probes, supporting the claim by the microarray manufacturer that longer probes are a better design strategy for increased signal strength. The expression patterns indicated by the signals were in good congruence with the annotation for these genes, and there was a positive correlation between our results and previously published [19] tiling microarray results (Fig. S3). Furthermore, the premier feature of high resolution for these two tiling arrays enabled differentiation between highly homologous family members, for example *SPL2* and *SPL10*, having high nucleotide sequence similarity (44.7% identity, Figure S2E and F; data not shown). Taken together, these results validated the chip design and the sensitivity of the method to detect transcript topology.

Another approach to assess the quality of microarray data is to reproduce the results of similar experiments performed by commercially available ATH1 microarray with the same mutants, viz. *hen1, dcl1, hyl1* and *rdr6* [16,19]. The pleiotropic nature of miRNA metabolism mutants is due in part to differential effects on specific miRNAs and developmental modulation of miRNA activities and/or abundance [16,21,80]. For example, miR160 accumulates in *dcl1-7* [37] while all other miRNAs are decreased to varying degrees, which in turn (but not always) results in increased expression of the corresponding miRNA targets [16,19]. For the 22 miRNA targets queried on our custom microarray, around 73% showed up-regulation on the sense strand in *hen1-1*. Most of the probes detected transcript abundances similar to published results reported for the same miRNA pathway mutants (Figs. S5- S16). There was a positive correlation of our *hen1-1* results (Pearson R^2^ = 0.31) for sense strand accumulation of target transcripts to those previously reported (Fig. S3) [19]. Interestingly, we also observed increased sense transcript accumulation in the *sgs3-14* mutant for several targets (*APS1*, *GRF8*, *TOE2*, *SPL10*, *At5g43740*/CC-*NBS*-*LRR*)(Table S7; Figs. S5, S8, S10, S12, S16), consistent with the report of Wu *et al*. [21] that *SPL4* transcripts were elevated in *rdr6-11* and *sgs3-11.* However, in contrast to other reports [16,19], we also observed down-regulation of sense transcripts in *dcl1-7* for *SPL10* (Fig. S12). Because *SPL* transcripts are developmentally regulated by temporal regulation of miR156 levels during vegetative phase change [21] and our experiments were carried out with RNA from flowers, tissue-specificity may account for these differences.
